# Turnover of EDEM1, an ERAD‐enhancing factor, is mediated by multiple degradation routes

**DOI:** 10.1111/gtc.13117

**Published:** 2024-04-29

**Authors:** Riko Katsuki, Mai Kanuka, Ren Ohta, Shusei Yoshida, Taku Tamura

**Affiliations:** ^1^ Department of Life Science, Graduated School of Engineering Science Akita University Akita Japan; ^2^ Department of Life Science, Faculty of Engineering Science Akita University Akita Japan; ^3^ Present address: Biococoon Laboratories Inc. 4‐3‐5, Ueda Morioka Japan

**Keywords:** autophagy, EDEM1, ER quality control, ER stress, ERAD, proteasome, protein degradation, protein turnover, unfolded protein response

## Abstract

Quality‐based protein production and degradation in the endoplasmic reticulum (ER) are essential for eukaryotic cell survival. During protein maturation in the ER, misfolded or unassembled proteins are destined for disposal through a process known as ER‐associated degradation (ERAD). EDEM1 is an ERAD‐accelerating factor whose gene expression is upregulated by the accumulation of aberrant proteins in the ER, known as ER stress. Although the role of EDEM1 in ERAD has been studied in detail, the turnover of EDEM1 by intracellular degradation machinery, including the proteasome and autophagy, is not well understood. To clarify EDEM1 regulation in the protein level, degradation mechanism of EDEM1 was examined. Our results indicate that both ERAD and autophagy degrade EDEM1 alike misfolded degradation substrates, although each degradation machinery targets EDEM1 in different folded states of proteins. We also found that ERAD factors, including the SEL1L/Hrd1 complex, YOD1, XTP3B, ERdj3, VIMP, BAG6, and JB12, but not OS9, are involved in EDEM1 degradation in a mannose‐trimming‐dependent and ‐independent manner. Our results suggest that the ERAD accelerating factor, EDEM1, is turned over by the ERAD itself, similar to ERAD clients.

## Graphical abstract

EDEM1 enhances endoplasmic reticulum‐associated degradation (ERAD). Here, we show the molecular detail of the EDEM1 turnover. We found that EDEM1 itself is degraded via ERAD by some ERAD‐related factors and also autophagy. The physiological reason for rapid EDEM1 turnover by ERAD is discussed.

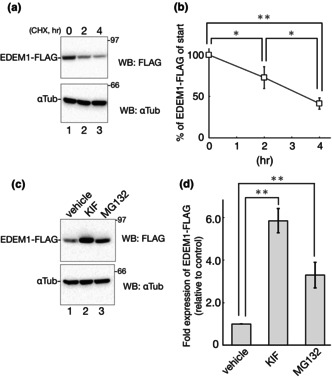

## 1 INTRODUCTION

The endoplasmic reticulum (ER) is a site for the production and disposal of secretory and membrane proteins, with stringent quality control checkpoints. This process is called the ER quality control (ERQC) system, which maintains ER function for the production of functional proteins and the degradation of misfolded proteins. During ER maturation, misfolded or misassembled proteins are subjected to the ER‐associated degradation (ERAD). ERAD consists of multiple steps for the disposal of terminally misfolded proteins: sorting from the folding machinery, delivery to the ER membrane for retrotranslocation, and polyubiquitination for degradation by the cytosolic proteasome (reviewed in Ninagawa et al., 2021).

When the ERQC system is defective or overwhelms its capacity, which is induced by fluctuations in the ER oxidative milieu, pathological conditions, or too many translated polypeptides loaded into the ER, it causes accumulation of incompletely folded proteins and ER stress (Wiseman et al., 2022). Cells react to ER stress by an unfolded protein response (UPR) to resolve protein issues. The UPR is equipped with three major signaling pathway branches that regulate ER protein levels and quality: attenuation of protein synthesis, protein refolding, ERAD, and apoptosis (Walter & Ron, 2011). Genetic regulation of the UPR and its clinical importance have been widely studied, but protein‐based regulation of the UPR is not well known. For example, after the removal of misfolded proteins and relief from ER stress, the mechanism by which overexpressed molecular chaperones and ERAD‐related proteins decrease to basal levels remains unclear.

ER degradation‐enhancing α‐mannosidase‐like protein 1 (EDEM1) is an early identified ERAD accelerating factor. Gene expression of EDEM1 is up‐regulated by ER stress and EDEM1 accelerates misfolded ERAD model substrates in a mannose‐trimming dependent manner (Hosokawa et al., 2001). Of the five N‐glycans of EDEM1, the first four are added co‐translationally, and the last C‐terminal unit is post‐translationally attached (Tamura et al., 2011). One of the characteristic features of EDEM1 is post‐translational signal sequence cleavage due to the inclusion of arginine residue within the transmembrane region, so that EDEM1 exists as both soluble and type II transmembrane proteins suitable for association with ERAD factors and substrates (Tamura et al., 2011). Over the past two decades, several studies have reported that EDEM1 enhances the ERAD of misfolded ER proteins. EDEM1 contains a mannosidase‐like domain, and this region exhibits mannose trimming activity in EDEM1 overexpressed cells (Hosokawa et al., 2010; Lamriben et al., 2018; Olivari et al., 2006) or upon incubation of immunoisolated EDEM1 with the Man8B N‐glycan (George et al., 2021; Shenkman et al., 2018). EDEM1 recognizes immature, but not mature, proteins through the free thiol and hydrophobic regions of misfolded proteins (Cormier et al., 2009; Lamriben et al., 2018). In addition, EDEM1 associates with SEL1L, the adaptor and receptor of the Hrd1 retrotranslocation complex (Chiritoiu et al., 2020; Cormier et al., 2009; Manica et al., 2021; Saeed et al., 2011; Tang et al., 2014), and this interaction is mediated in a mannose trimming‐dependent manner (Cormier et al., 2009; Saeed et al., 2011). However, the regulation of the EDEM1 biosynthesis and degradation machinery is not well defined. Basal‐level autophagy, but not starvation‐induced autophagy, has been reported to be involved in the turnover of EDEM1 (Calì et al., 2008). Several studies have shown that autophagy (Le Fourn et al., 2009; Park et al., 2014) and the ERAD (Chiritoiu et al., 2020) execute the EDEM1 turnover. To better understand the entire ERQC mechanism and the turnover of UPR‐related proteins, the molecular mechanism of EDEM1 clearance at the protein level should be clarified.

We investigated the fate of EDEM1 in tissue culture cell lines using exogenous EDEM1 expression. We found that EDEM1 turnover was delayed by the inhibition of ER mannosidase I or proteasome activity, similar to misfolded ERAD client proteins. Analysis of detergent‐insoluble protein aggregates indicated that ERAD and autophagy may target different folding states of EDEM1. We revealed that YOD1, a deubiquitinase critical for the retro‐translocation of ERAD substrates by trimming the polyubiquitin chain, strongly participates in EDEM1 clearance. We also found that Hrd1, one of the major E3 ubiquitin ligases for ERAD substrates, and SEL1L, which accepts ERAD substrates and accessory proteins, were involved in EDEM1 degradation. Furthermore, several ERAD‐related factors, including XTP3B, ERdj3, BAG6, VIMP, and JB12, but not OS9, facilitate EDEM1 degradation in a mannose trimming‐dependent or ‐independent manner. Our findings support the notion that the half‐life of EDEM1 is regulated by the proteasome through the ERAD pathway, similar to that of misfolded ERAD substrates. The necessity and physiological role of rapid EDEM1 turnover in response to ER stress are discussed.

## 2 RESULTS

### 2.1 EDEM1 is rapidly turned over by ERAD

**FIGURE 1:**
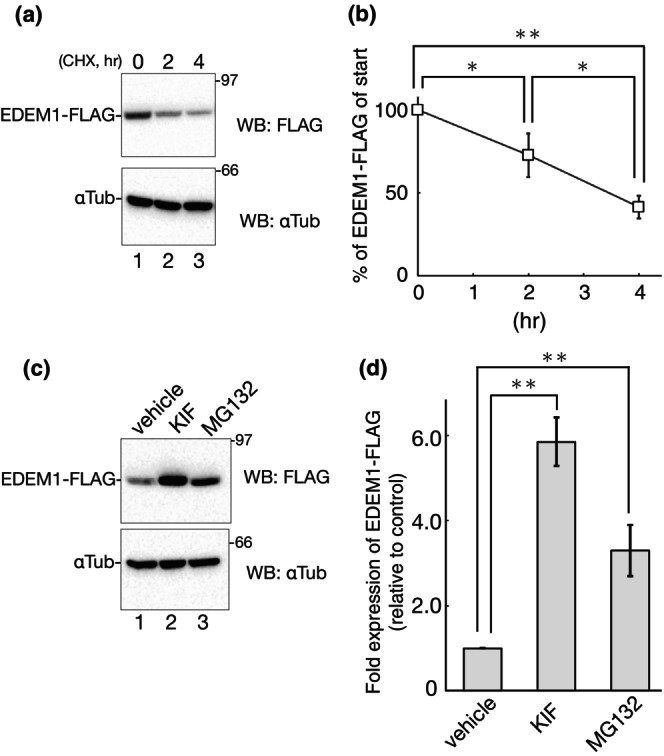
EDEM1 degradation by ERAD. (a) 293 EBNA cells were transfected with EDEM1‐FLAG then treated with 10 μg/mL CHX for the indicated time. αTubulin was used as the loading control. Western blotting was performed with the indicated antibodies. (b) Protein bands in (a) were quantified and plotted by the three independent experiments. The EDEM1‐FLAG band intensity was normalized by dividing it by that of αTubulin, and time 0 was as 100% by the three independent experiments. *P < 0.05; **P < 0.01 (two‐tailed Student's t‐test). (c) Transfected 293 EBNA cells with EDEM1‐FLAG were treated with indicated drug (150 μM KIF or 1 μM MG132) for 16 h, respectively. Western blotting was performed with the indicated antibodies under reducing conditions. (d) The quantification of (c). The EDEM1‐FLAG band intensity was normalized by dividing it by that of αTubulin, and vehicle treatment was regarded as 1.0 by the three independent experiments. **P < 0.01 (two‐tailed Student's t‐test).

To obtain details of the EDEM1 turnover process, we exogenously expressed C‐terminal FLAG‐tagged EDEM1 in 293 EBNA cells and evaluated the expression level of EDEM1‐FLAG. The EDEM1 turnover rate was analyzed by the time‐course of cycloheximide (CHX) treatment in which cellular protein synthesis was inhibited, and the remaining proteins were detected by western blotting at each time point. The CHX‐chase experiments showed that EDEM1‐FLAG exhibited a turnover rate of approximately 3 h (Figure 1a,b). This result is consistent with previous reports (Calì et al., 2008; Lamriben et al., 2018; Le Fourn et al., 2009; Tamura et al., 2011), regardless of whether endogenous or exogenously expressed EDEM1 was detected by CHX‐chase or metabolic pulse‐chase radiolabeling.

To ascertain whether the degradation of EDEM1 is mediated by ERAD, cells were incubated with the ERAD inhibitory drugs kifunensine (KIF) and MG132. KIF suppresses ER mannosidase I and prevents mannose‐trimming of glycoproteins from Man9 to Man8, 5–6 in the ER, whereas MG132 is a peptide analog that disturbs proteases of the proteasome, both of which are widely used inhibitors of ERAD, although KIF is limited to ERAD of N‐glycosylated substrate proteins. During the 48 h transfection, each drug was incubated for the last 16 h, and the expression level of EDEM1‐FLAG was inspected by western blotting. KIF and MG132 clearly recovered EDEM1‐FLAG expression levels (Figure 1c,d), as shown previously (Tamura et al., 2011). These results demonstrate that these drugs work under our experimental conditions and are consistent with a previous report that EDEM1‐FLAG is degraded through ERAD (Chiritoiu et al., 2020).

**FIGURE 2:**
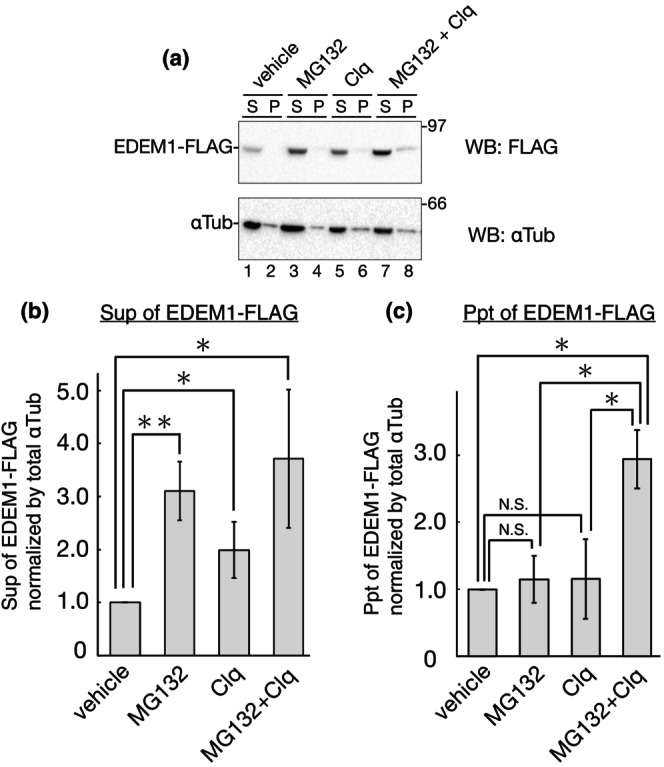
Targeting of EDEM1‐FLAG aggregates by autophagy. (a) 293 EBNA cells were transfected with EDEM1‐FLAG and treated with indicated drug (1 μM MG132, 50 μM Clq, or both) for 16 h and cell lysates were separated to detergent‐soluble (S) and detergent‐insoluble fractions (P). Samples were resolved in reducing SDS‐PAGE and immunoblotted using anti‐FLAG and anti‐αTubulin antibodies. (b) Quantification of the supernatant (S) fraction of A (lanes 1, 3, 5, and 7). EDEM1‐FLAG expression was normalized to that of αTub. A vehicle‐treated sample was regarded as 1.0 by the four independent experiments. *P < 0.05; **P < 0.01 (two‐tailed Student's t‐test). (c) Quantification of precipitation (P) fraction of A (lanes 2, 4, 6, and 8) was conducted as in (b) by the three independent experiments. *P < 0.05; N.S., not significant (two‐tailed Student's t‐test).

To verify the mobility shift generated by KIF is due to the remaining mannoses, N‐glycans of EDEM1‐FLAG were removed by PNGaseF treatment, and following western blotting was conducted. KIF‐treated EDEM1‐FLAG migrated slower than the vehicle in mock‐treated samples (Figure S1a, lanes 1 and 2). Interestingly, KIF‐treated and de‐glycosylated EDEM1‐FLAG still migrated slower (Figure S1a, lanes 3 and 4). The combination of MG132 and Clq treatment retards degradation of EDEM1‐FLAG aggregates (Figure 2a, lane 8) and following PNGaseF treatment showed slower migration of EDEM1‐FLAG in the detergent‐insoluble fraction (Figure S1b). Since KIF treatment persists association of EDEM1 with ER lectin chaperone calnexin and misfolded ERAD substrate proteins (Molinari et al., 2003), KIF could lead to a delay of the EDEM1 signal sequence cleavage by a steric hindrance. Our preliminary results also showed that chemically induced ER stress suppresses signal sequence cleavage of EDEM1‐FLAG (data not shown). Although EDEM1‐FLAG keeps detergent solubility by KIF (Figure S2a), long‐term KIF treatment (16 h) could cause accumulation of EDEM1‐FLAG in the ER with an uncleaved signal sequence or unknown post‐translational modifications.

### 2.2 Autophagy targets aggregated EDEM1 generated by the proteasome suppression

At first, it has been showed that EDEM1 is degraded by basal level but not starvation‐induced autophagy (Calì et al., 2008). To differentiate between ERAD and autophagy in EDEM1 clearance, we inhibited autophagy using chloroquine (Clq), a lysosome inhibitor, and fractionated the cell lysate into detergent‐soluble and detergent‐insoluble fractions to analyze the aggregates based on protein hydrophobicity. Clq treatment recovered EDEM1‐FLAG expression similar to MG132 treatment in detergent‐soluble fractions (Figure 2a, lanes 1, 3, and 5; Figure 2b). In the vehicle treatment, EDEM1‐FLAG was almost undetectable in the detergent‐insoluble fraction (Figure 2a, lane 2), suggesting that majority of EDEM1‐FLAG in this condition is expressed as soluble but not an aggregate. And by either drug treatment, EDEM1‐FLAG in the detergent‐insoluble fraction was not significantly changed (Figure 2a, lanes 2, 4, and 6; quantification is shown in Figure 2b). However, simultaneous treatment with MG132 and Clq significantly increased detergent‐insoluble EDEM1‐FLAG (Figure 2a, lane 8; quantification is shown in Figure 2c). Another autophagy suppressor, wortmannin (WM), a widely used inhibitor of phosphatidylinositol 3 kinase that prevents autophagosome formation, showed similar results to Clq; a single treatment with WM only recovered soluble EDEM1‐FLAG, whereas EDEM1‐FLAG aggregates were detected by both WM and MG132 treatment (Figure S2b). These results suggest that autophagy targets aggregated EDEM1‐FLAG induced by proteasome inhibition rather than soluble EDEM1‐FLAG. These effects of drugs in EDEM1‐FLAG degradation were also confirmed by the CHX‐chase experiments (Figure S3). KIF treatment did not suppress the degradation of EDEM1‐FLAG (Figure S3a, lanes 3 and 5). This result suggests that the majority of mannose residues in EDEM1‐FLAG detected by western blotting after 48 h transfection were already trimmed to Man5–7 from Man9. EDEM1‐FLAG detected in the detergent‐insoluble fraction migrated slower than that in the detergent‐soluble fraction (Figure 2a, lanes 7 and 8; Figure S2b, lanes 5 and 6). This change was observed after N‐glycan removal by PNGase F treatment (Figure S1b), suggesting that the band shift between detergent‐soluble and insoluble EDEM1‐FLAG was probably due to the existence of the transmembrane region of EDEM1 or unknown post‐translational modification (see Figure S1a) rather than N‐glycosylation.

### 2.3 EDEM1 is accumulated in the ER under ERAD inhibition by KIF treatment

**FIGURE 3:**
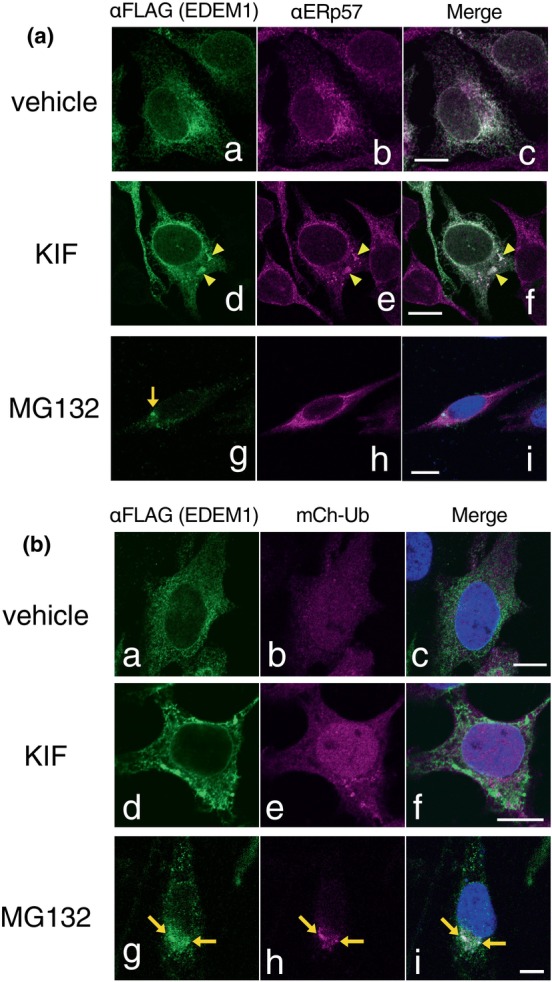
Cellular location of EDEM1‐FLAG under ERAD inhibition. (a) Transfected HeLa cells were treated with vehicle (a–c), 150 μM KIF (d–f), or 1 μM MG132 (g–i). After fixation and permeabilization, indirect immunofluorescence was performed using anti‐FLAG (green; a, d, g) and anti‐ERp57 (magenta; b, e, h) antibodies. Merged images of representative cells (c, f, and i) are presented. Scale bars, 10 μm. (b) Co‐transfection of HeLa cells with mCherry‐Ub and EDEM1‐FLAG. Cells were treated with vehicle (a–c), 150 μM KIF (d–f), or 1 μM MG132 (g–i). Indirect immunofluorescence experiments were performed using anti‐FLAG antibodies (green, a, d, and g). Representative cells with mCherry‐Ub signals (b, e, and h) and merged images (c, f, and i) are shown. Scale bars, 10 μm.

To further elucidate the cellular location of EDEM1, we expressed EDEM1‐FLAG in HeLa cells and visualized EDEM1‐FLAG under ERAD inhibition by indirect immunofluorescence staining followed by confocal microscopy. Exogenously expressed EDEM1‐FLAG was colocalized with ERp57, a soluble ER‐resident chaperone, indicating that EDEM1‐FLAG was located in the ER (Figure 3a, a–c). Under KIF treatment, EDEM1‐FLAG exhibited an aggregate‐like structure, which colocalized with ERp57 (Figure 3a, d–f) and other ER‐resident chaperones, BiP and CRT (Figure S4). These results suggest that the inhibition of ERAD by KIF retains EDEM1‐FLAG in the ER lumen and is thereby recognized by chaperones, probably because of the instability of EDEM1. KIF generated only negligible detergent‐insoluble EDEM1‐FLAG in the fractionation and subsequent immunoblotting experiments (Figure S3a). During KIF treatment, a small amount of cellular ubiquitinated proteins accumulated (Figure 3b, compare panels b and e), but EDEM1‐FLAG did not colocalize with mCherry‐tagged ubiquitin (Figure 3b, d–f). The results indicate that the inhibition of ERAD by KIF causes EDEM1‐FLAG accumulation in the ER, but not in the cytosol. In the case of proteasome inhibition by MG132, EDEM1‐FLAG also accumulated but did not colocalize with ERp57 (Figure 3a, g–i). EDEM1‐FLAG was located next to the nucleus and colocalized with mCherry‐Ub in some cells (Figure 3b, g–i), suggesting the transport of EDEM1‐FLAG to the aggresome after retrotranslocation and polyubiquitination. Notably, not all EDEM1‐FLAG in co‐transfected cells overlapped with mCherry‐Ub under proteasome inhibition; 18% of EDEM1‐FLAG was completely colocalized with mCherry‐Ub, 32% of EDEM1‐FLAG was colocalized with mCherry‐Ub and remained in the ER, and half of EDEM1‐FLAG was found only in the ER under our experimental condition (Figure S5).

### 2.4 Deubiquitinase activity of YOD1 is critical for EDEM1 degradation

**FIGURE 4:**
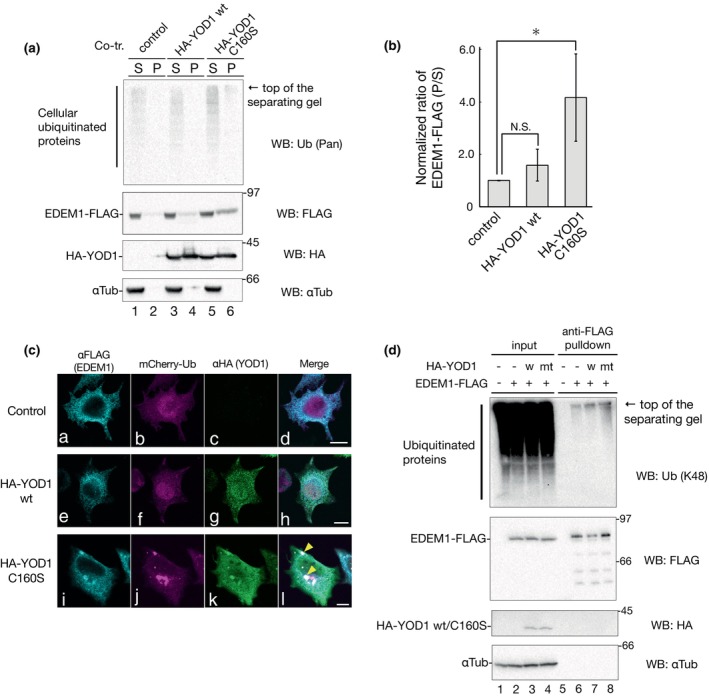
YOD1 is critical for EDEM1‐FLAG degradation. (a) 293 EBNA cells were transfected with EDEM1‐FLAG and empty vector, HA‐YOD1 WT, or HA‐YOD1 C160S. Cell lysates were fractionated into detergent‐soluble (S) and detergent‐insoluble (P) fractions. After resolution by reducing the SDS‐PAGE gel, the proteins of interest were detected using the indicated antibodies. (b) Quantification of EDEM1‐FLAG of (a). Supernatant and precipitation of EDEM1‐FLAG in (a) were quantified and the ratio normalized with αTubulin are shown. Samples of the control vector (lanes 1 and 2) were regarded as 1.0 by the four independent experiments. *P < 0.05; N.S., not significant (two‐tailed Student's t‐test). (c) Cotransfection and indirect immunofluorescence of HeLa cells. HeLa cells were transfected with EDEM1‐FLAG, mCherry‐Ub, control vectors (a–d), HA‐YOD1 WT (e–h), or HA‐YOD1 C160S (i–l). Immunostaining experiments using anti‐FLAG (turquoise, 647 nm) and anti‐HA (green, 488 nm) antibodies were performed and visualized using an mCherry‐Ub signal (magenta). Merged images (d, h, and l) are shown and scale bars, 10 μm. (d) 293 EBNA cells were transfected with an empty vector (lanes 1 and 5), EDEM1‐FLAG (lanes 2 and 6), EDEM1‐FLAG with HA‐YOD1 WT (lane 3 and 7, as WT), or EDEM1‐FLAG with HA‐YOD1 C160S (lanes 4 and 8, as mt). Detergent‐soluble lysates were separated and used as inputs for lysates (lanes 1–4) or anti‐FLAG pull‐down (lanes 5–8). After reducing SDS‐PAGE, the proteins of interest were detected using the indicated antibodies.

During the retrotranslocation of misfolded proteins from the ER to the cytosol, trimming of the polyubiquitin chain by the deubiquitinase YOD1 is important for p97 action, where a proper length of the polyubiquitin chain is needed (Ernst et al., 2009). To confirm that trimming of the polyubiquitin chain by YOD1 is required for EDEM1 degradation by ERAD, similar to misfolded proteins, we employed WT and enzymatically inactive forms of YOD1 (C160S). As shown in Figure 4a, HA‐tagged YOD1 C160S increased cellular ubiquitinated proteins both in detergent‐soluble and detergent‐insoluble fractions (Lanes 5 and 6), consistent with previous reports (Ernst et al., 2009; Locke et al., 2014), YOD1 C160S exhibited a dominant‐negative effect on cellular ubiquitinated proteins. Under our experimental conditions, EDEM1‐FLAG appeared strongly in the detergent‐insoluble fraction upon co‐expression with YOD1 C160S (Figure 4a, lanes 5 and 6; quantification shown in Figure 4b). These results suggest that the trimming of the polyubiquitin chain by YOD1 is required for proper EDEM1 turnover. In fact, EDEM1‐FLAG colocalized with ubiquitin in part when co‐expression with YOD1 C160S as aggregates, as evaluated by indirect immunofluorescence (Figure 4c, panels i–l). Furthermore, polyubiquitinated EDEM1‐FLAG was increased by YOD1 C160S compared to cotransfection with the control vector or WT YOD1, as verified by pull‐down and subsequent immunoblotting using anti‐K48 ubiquitin (Figure 4d, lane 8). Collectively, these results suggest that EDEM1 turnover is mediated by ERAD through polyubiquitination and trimming process by YOD1.

### 2.5 The SEL1L and Hrd1 complex is involved in EDEM1 clearance

Next, we investigated whether ERAD factors were involved in EDEM1 clearance through the ERAD pathway. One of the retrotranslocation pathways coupled with polyubiquitination by E3 in ERAD is the SEL1L/Hrd1 complex. Hrd1, an E3 ubiquitin ligase, is a multimembrane spanning channel and creates the space for passage of misfolded proteins from the ER (Bhattacharya et al., 2022; Iida et al., 2011; Wu et al., 2020). SEL1L acts as a receptor for soluble transmembrane adaptor proteins such as XTP3B, OS9, EDEM1, and Hrd1 (Christianson et al., 2008; Cormier et al., 2009; Iida et al., 2011).

**FIGURE 5:**
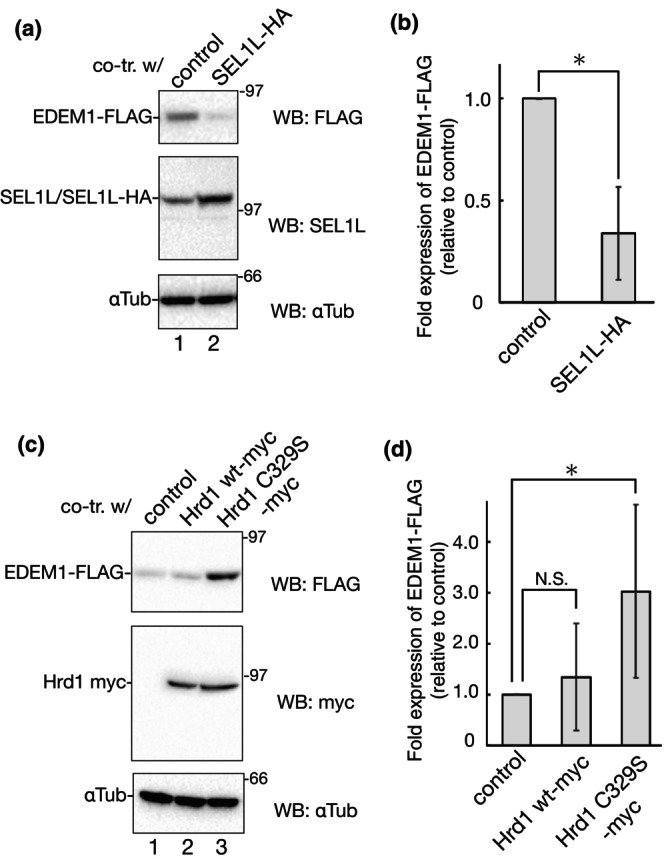
Involvement of SEL1L/Hrd1 in EDEM1‐FLAG degradation. (a) 293 EBNA cells were transfected with EDEM1‐FLAG and empty vector, or SEL1L‐HA. Cell lysates were subjected to reducing SDS‐PAGE and western blotting with the indicated antibodies. (b) Quantification of (a). Bands of EDEM1‐FLAG in (a) were quantified and the ratio was normalized with αTubulin. Samples of the control vector were regarded as 1.0 by the three independent experiments. *P < 0.05 (two‐tailed Student's t‐test). (c) Transfection of 293 EBNA cells with EDEM1‐FLAG and empty vector, Hrd1 WT‐myc, or Hrd1 C329S‐myc. After reducing SDS‐PAGE of the cell lysates, western blotting was performed using the indicated antibodies. (d) Quantification of (c). Bands of EDEM1‐FLAG in (c) were quantified and the ratio was normalized with αTubulin. Samples of the control vector were regarded as 1.0 by the six independent experiments. *P < 0.05; N.S., not significant (two‐tailed Student's t‐test).

Overexpression of C‐terminal HA‐tagged SEL1L clearly down regulates EDEM1‐FLAG (Figure 5a,b). We also confirmed that the expression level of SEL1L affected EDEM1‐FLAG degradation by siRNA knockdown in SEL1L and CHX‐chase experiments (Figure S6). Previously, we have shown that EDEM1 binds to SEL1L in a mannose trimming‐dependent manner (Cormier et al., 2009; Tamura et al., 2011). Since EDEM1 degradation was significantly suppressed by KIF treatment (Figure 1c), SEL1L overexpression increased the chances of EDEM1 binding to SEL1L and subsequent degradation through the SEL1L/Hrd1 pathway. Otherwise, SEL1L enhances EDEM1‐FLAG degradation independent of Hrd1, because overexpressed SEL1L is detected as a Hrd1‐free form that is degraded by the proteasome (Iida et al., 2011).

We evaluated whether E3 Hrd1 is involved in EDEM1 degradation. Coexpression of myc‐tagged WT Hrd1 did not show a significant effect on EDEM1 protein expression level, whereas the E3 inactive mutant, Hrd1 C329S, which lacks polyubiquitination activity (Morito et al., 2008), recovered EDEM1‐FLAG (Figure 5c,d). Interestingly, CRISPR/Cas9‐based knockout of SEL1L in HEK293 cells (van der Goot et al., 2018), tamoxifen‐induced SEL1L knockout in the mouse pancreas (Sun et al., 2014), or both siRNA of SEL1L and Hrd1 in HEK293T up‐regulates EDEM1 protein expression (Chiritoiu et al., 2020). These reports are consistent with our results: SEL1L facilitates EDEM1 degradation, whereas the E3‐inactive form of Hrd1 negatively affects EDEM1 turnover, suggesting that the SEL1L/Hrd1 pathway is a crucial route for EDEM1 degradation.

### 2.6 Differential effect of ERAD lectins, XTP3B, and OS9, in EDEM1 turnover

Misfolded proteins destined for ERAD are subjected to post‐translational modifications, in which mannoses of N‐glycans on ERAD substrates are trimmed to 5–6 mannoses by ER Mannosidase I or EDEM1‐3 proteins (George et al., 2021; Hosokawa et al., 2003). The ER‐resident lectins OS9 and XTP3B can bind 5–7 mannose trimmed N‐glycans in the mannose 6‐phosphate receptor homology (MRH) domain (Hosokawa et al., 2009; Satoh et al., 2010; Yamaguchi et al., 2009). OS9 and XTP3B are strongly involved in ERAD through interaction with ERAD substrates (Hosokawa et al., 2009; Yamaguchi et al., 2009), the N‐glycan of SEL1L (Christianson et al., 2008), or both (van der Goot et al., 2018). Therefore, we evaluated the functional roles of XTP3B and OS9 in EDEM1‐FLAG degradation.

**FIGURE 6:**
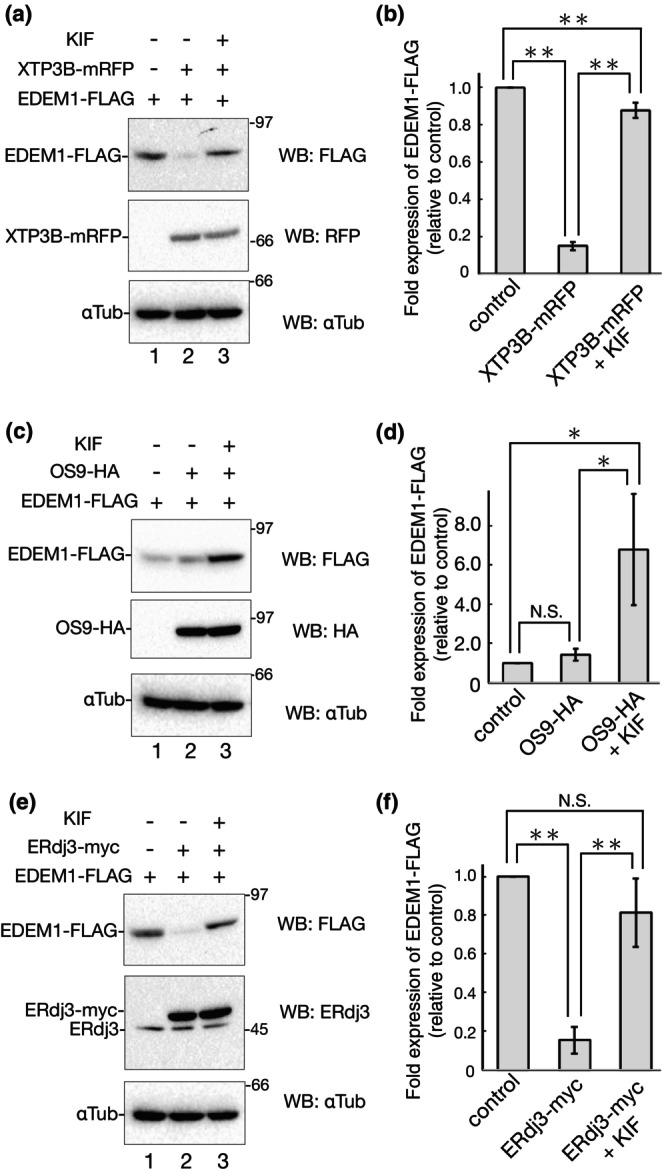
Differential effect of ERAD lectins XTP3B and OS9 and ERdj3 in EDEM1‐FLAG degradation. (a) 293 EBNA cells were transfected with EDEM1‐FLAG and empty vector or XTP3B‐mRFP with or without 150 μM KIF for 16 h (indicted at the top of the blot). Cell lysates were subjected to reducing SDS‐PAGE and western blotting with the indicated antibodies was performed. (b) Quantification of (a). Bands of EDEM1‐FLAG in (a) were quantified and the ratio was normalized with αTubulin. Samples of the control vector were regarded as 1.0. Data were compared using a two‐tailed Student's *t*‐test by the four independent experiments. **P < 0.01. (c) 293 EBNA cells were transfected with EDEM1‐FLAG and empty vector or OS9‐HA with or without 150 μM KIF for 16 h (indicted at the top of the blot). Western blotting was performed as described in (a), except for the use of anti‐HA for OS9‐HA. (d) Quantification of (c) by the four independent experiments. Data were treated as described in (b). *P < 0.05; N.S., not significant. (e) Transfected 293 EBNA cells with EDEM1‐FLAG and empty vector or ERdj3‐myc with or without 150 μM KIF for 16 h (indicted at the top of the blot). After reducing SDS‐PAGE, western blotting was performed using the indicated antibodies. (f) Quantification of (e). Bands of EDEM1‐FLAG in (e) were quantified and the ratio was normalized with αTubulin. Samples of the control vector were regarded as 1.0. Data were compared using a two‐tailed Student's *t*‐test by the four independent experiments. **P < 0.01; N.S., not significant.

Coexpression of XTP3B (isoform 1) fused with mRFP at the C‐terminus significantly down‐regulates EDEM1‐FLAG (Figure 6a, lanes 1 and 2). Interestingly, this decrease was reversed upon incubation with KIF (Figure 6a, lanes 2 and 3), suggesting that XTP3B facilitates EDEM1‐FLAG degradation through the mannose trimming‐mediated ERAD pathway. In contrast, HA‐tagged OS9 (variant 2) at the C‐terminus did not affect the EDEM1‐FLAG turnover (Figure 6c, lanes 1 and 2) or KIF treatment (Figure 6c, lanes 1 and 3). EDEM1‐FLAG physically interacted with OS9‐HA, as elucidated by co‐immunoprecipitation experiments (Figure S7), as reported by Chiritoiu et al. (2020). It seems that this OS9 association is not required for EDEM1‐FLAG turnover but may be needed for ERAD enhancement as a functional complex. Collectively, our results show a contrasting effect of OS9 and XTP3B. This distinction could reflect the substrate specificity of XTP3B and OS9 for EDEM1‐FLAG degradation.

### 2.7 ERdj3 facilitates EDEM1 degradation in a KIF‐dependent manner

Next, we used ERdj3, a well‐characterized DnaJ domain‐containing protein that acts as a BiP cochaperone in the ER (Shen & Hendershot, 2005). ERdj3 binds directly to misfolded proteins and acts as a BiP cochaperone for refolding (Shen & Hendershot, 2005; Tan et al., 2014). The protein expression level of EDEM1‐FLAG was monitored by cotransfection with myc‐tagged ERdj3 at the C‐terminus. ERdj3‐myc overexpression clearly enhanced EDEM1‐FLAG degradation (Figure 6e, lanes 1 and 2). Interestingly, this effect was suppressed by KIF (Figure 6e, lanes 1–3), indicating that ERdj3 is involved in the ERAD of EDEM1‐FLAG in a mannose trimming‐dependent manner. ERdj3 recruits misfolded proteins to BiP through its J domain, which is required for BiP association (Shen & Hendershot, 2005). To clarify whether EDEM1 degradation by ERdj3 is mediated by BiP, we introduced the point mutation into the J domain of ERdj3 and monitored EDEM1 degradation by co‐expression. Mutation of the histidine residue in the J domain to glutamine abolished the interaction between ERdj3 and BiP (Figure S8a, lanes 11 and 12). Co‐expression of ERdj3 J domain mutant, H53Q, showed a similar decrease effect in EDEM1‐FLAG degradation compared to WT (Figure S8b). In these experiments, the cellular expression level of H53Q mutant was lower than the WT (Figure S8a, lanes 2 and 3; Figure S8b, lanes 2 and 3). ERdj3 does not contain an ER retrieval signal such as KDEL sequence and secretion of ERdj3 into the culture medium has been reported (Genereux et al., 2015; Hanafusa et al., 2019). Therefore, we examined the protein secretion of ERdj3 WT and H53Q and we found that the secretion of ERdj3 H53Q is much higher than that of WT (Figure S8c, lanes 5 and 6). This difference is probably explained by the lack of BiP tethering in the ER lumen for ERdj3 H53Q. Alternatively, ERdj3 may act as a molecular chaperone to enhance the degradation of EDEM1 through its interaction with SDF2 (Hanafusa et al., 2019).

### 2.8 Cytosolic BAG6 and VIMP enhance EDEM1 degradation through ERAD

**FIGURE 7:**
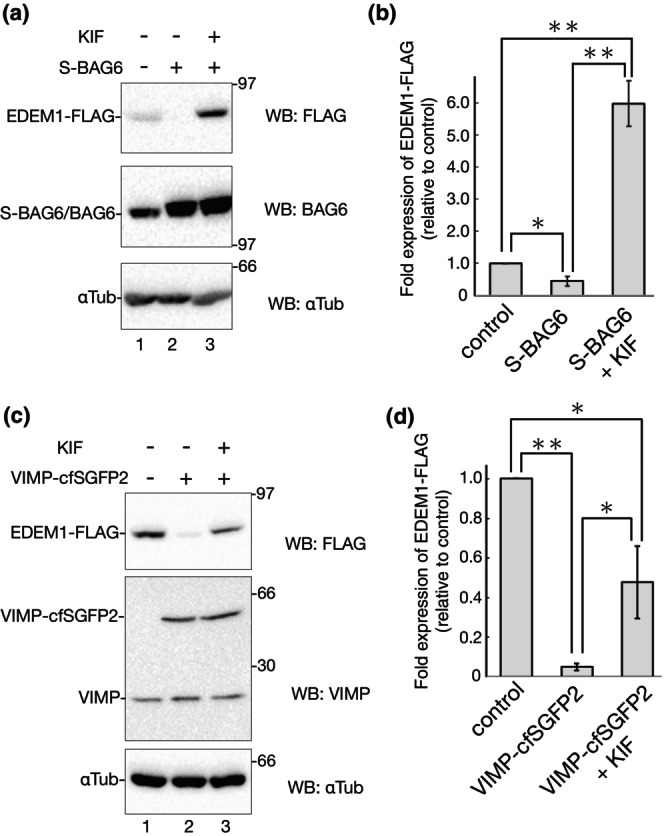
Involvement of cytosolic ERAD factors BAG6 and VIMP in EDEM1‐FLAG degradation in a KIF‐dependent manner. (a) Transfected 293 EBNA cells with EDEM1‐FLAG and empty vector or S‐BAG6 with or without 150 μM KIF for 16 h (indicted at the top of the blot). After reducing SDS‐PAGE, western blotting was performed using the indicated antibodies. (b) Quantification of (a). Bands of EDEM1‐FLAG in (a) were quantified and the ratio was normalized with αTubulin. Samples of the control vector were regarded as 1.0. Data were compared using a two‐tailed Student's *t*‐test by the three independent experiments. *P < 0.05; **P < 0.01. (c) Transfected 293 EBNA cells with EDEM1‐FLAG and empty vector or VIMP‐cfSGFP2 with or without 150 μM KIF for 16 h (indicted at the top of the blot). After reducing SDS‐PAGE, western blotting was performed using the indicated antibodies. (d) Quantification of (c) by the three independent experiments. Data were treated as (b). *P < 0.05; **P < 0.01.

In addition to ER luminal and membrane‐anchored factors, cytosolic ERAD‐related proteins are involved in ERAD by regulating p97 activity during retrotranslocation, polyubiquitination, deubiquitination, and targeting of clients to the proteasome (Christianson & Ye, 2014). BAG6 interacts with ERAD E3 ligases, including Hrd1, AMFR/gp78, and RMA1 (Wang et al., 2011; Zhang et al., 2015). Gp78, a multimembrane‐spanning E3 ligase, has been well characterized for its functional relationship with BAG6 in the retrotranslocation process (Wang et al., 2011; Xu et al., 2012; Zhang et al., 2015). BAG6 forms a functional complex with gp78 and p97 for the retrotranslocation of misfolded proteins from the ER to the cytosol (Xu et al., 2012; Zhang et al., 2015). We evaluated the role of BAG6 in EDEM1 turnover following BAG6 overexpression. Cotransfection with S‐tagged BAG6 effectively decreased EDEM1‐FLAG expression (Figure 7a, lanes 1 and 2). This reduction in EDEM1‐FLAG was significantly reversed by KIF treatment (Figure 7a, lane 3), suggesting that mannose trimming is required for the degradation of EDEM1‐FLAG by the BAG6‐gp78 pathway. Compared to XTP3B and ERdj3 (Figure 6), the recovery rate of KIF with BAG6 overexpression was higher and almost equivalent to that of KIF treatment (Figures 1c and 7b). As KIF treatment resulted in ER retention of EDEM1‐FLAG (Figure 3), this effect occurred because BAG6, which enhances retrotranslocation through p97 and gp78, had little access to EDEM1‐FLAG under KIF treatment due to its cytosolic localization.

We also verified the role of another cytosolic ERAD‐related factor, VIMP, in EDEM1 degradation. VIMP is part of the retrotranslocation complex with Derlin1 (Eura et al., 2020; Ye et al., 2004), Derlin2 (Huang et al., 2013; Lilley & Ploegh, 2005), and ERAD‐related E3s, including RMA1 (Hou et al., 2018), TMEM129 (Van De Weijer et al., 2014), and gp78 (Ballar et al., 2007; Zhong et al., 2011), but not Hrd1. VIMP facilitates degradation of CFTRΔ508 (Hou et al., 2018) or MHC class I (Ye et al., 2004) in co‐operation with Derlin1 and p97. Furthermore, the interaction between VIMP and EDEM1 was revealed using a proteomic approach (Manica et al., 2021). We cotransfected 293 EBNA cells with EDEM1‐FLAG and VIMP‐cfSGFP2, fused with cysteine free SGFP2 at the C‐terminus, and examined the protein expression levels of EDEM1‐FLAG. As shown in Figure 7c,d, coexpression of VIMP clearly stimulates EDEM1‐FLAG degradation (Figure 7c, lanes 1 and 2). This decrease was dependent on KIF (Figure 7c, lane 1–3), but was not comparable in the case of BAG6 (Figure 7a,b). The reason for this difference is unknown; however, VIMP contains the ER luminal short‐tail C‐terminus, whereas BAG6 is a cytosolic protein (part of which exists in the nucleus). The C‐terminal tail of VIMP may be associated with factors in the KIF‐dependent ERAD pathway and exhibit a positive role in ERAD.

### 2.9 JB12 facilitates transmembrane form EDEM1 clearance in a KIF‐independent manner

**FIGURE 8:**
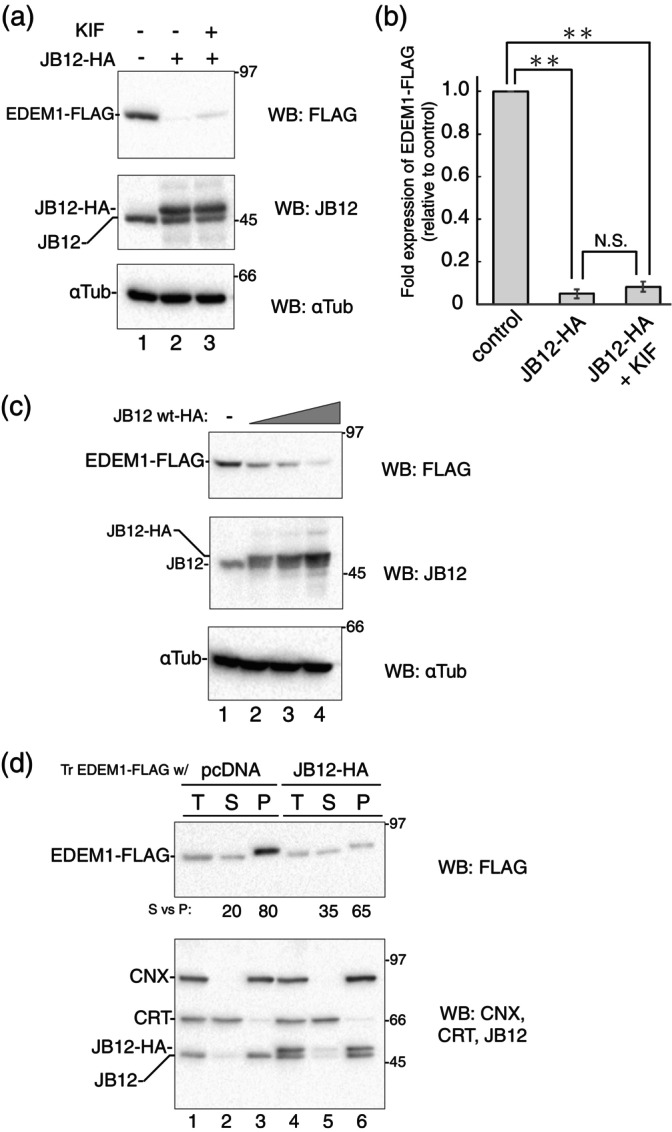
JB12 enhances degradation of transmembrane form of EDEM1‐FLAG degradation in a KIF‐independent manner. (a) Transfected 293 EBNA cells with EDEM1‐FLAG and empty vector or JB12‐HA (0.1 μg) with or without 150 μM KIF for 16 h (indicted at the top of the blot). After reducing SDS‐PAGE, western blotting was performed using the indicated antibodies. (b) Quantification of (a). Bands of EDEM1‐FLAG in (a) were quantified and the ratio was normalized with αTubulin. Samples of the control vector were regarded as 1.0. Data were compared using a two‐tailed Student's *t*‐test by the three independent experiments. **P < 0.01; N.S., not significant. (c) 293 EBNA cells were transfected with EDEM1‐FLAG and empty vector (lane 1) or JB12‐HA (lanes 2–4), with an increased amount of the JB12‐HA expressing vector (0.02, 0.05, and 0.1 μg). After reducing SDS‐PAGE, western blotting was performed using the indicated antibodies. (d) Alkaline extraction and fractionation of EDEM1‐FLAG. Transfected 293 EBNA cells with EDEM1‐FLAG and pcDNA or JB12‐HA (0.02 μg) were homogenated and the microsomes from the post‐nuclear supernatant were obtained as described in Section 4. Microsomes were treated with an alkaline buffer (total sample, T) and fractionated into soluble (supernatant, S) and membrane‐anchored (pellet, P) proteins. After reducing SDS‐PAGE, western blotting was performed using anti‐FLAG (top panel) and a mixture of anti‐CNX, ‐CRT, and ‐JB12 (bottom panel). Ratio of EDEM1‐FLAG determined in the S and P fractions is shown.

The ER‐localized DnaJ heat shock protein 40 family member B12 (JB12) preferentially enhances the ERAD of misfolded transmembrane proteins rather than soluble proteins (Grove et al., 2011; Yamamoto et al., 2010). As comprehensive analyses have shown an association between JB12 and EDEM1 (Chiritoiu et al., 2020; Manica et al., 2021), we investigated whether these interactions are involved in EDEM1 turnover. Coexpression of HA‐tagged JB12 increased the degradation of EDEM1‐FLAG (Figure 8a,b). This reduction was dependent on JB12‐HA expression (Figure 8c), suggesting that JB12 promotes EDEM1 degradation. Interestingly, this degradation was not recovered by KIF treatment (Figure 8a, lanes 2 and 3), suggesting mannose trimming‐independent degradation of EDEM1‐FLAG. This JB12‐assisted degradation was slightly inhibited by Clq but not WM, suggesting that the lysosome but not autophagy is involved in EDEM1‐FLAG degradation (Figure S9a,b). The secretion of EDEM1‐FLAG into the media was not detected (Figure S9a, lane 13). JB12 has a cytosolic J domain that enables collaboration with the chaperones Hsc70 or HSP70 to recruit the ER quality control region (Grove et al., 2011). We employed the J domain mutant, H138Q, which prevents the binding of JB12 to cytosolic chaperones. This JB12 mutant showed similar EDEM1‐FLAG clearance to that of WT JB12, despite lacking cytosolic chaperone‐binding ability (Figure S9c,d), indicating that Hsc70/HSP70 was not involved in JB12‐mediated EDEM1‐FLAG degradation.

We noticed that the migration of EDEM1‐FLAG during electrophoresis was faster when JB12‐HA was coexpressed (Figure 8c, lanes 1–4). Since JB12 prefers membrane‐anchored ERAD substrates over soluble proteins (Grove et al., 2011; Yamamoto et al., 2010), it is possible that JB12 selectively targets the transmembrane form of EDEM1‐FLAG. The signal sequence of EDEM1 is post‐translationally cleaved so that EDEM1 exists as both ER luminal and a type II transmembrane protein (Tamura et al., 2011), the changes of EDEM1‐FLAG migration reflect the involvement of JB12 in specific degradation of membrane‐anchored EDEM1‐FLAG. We employed alkaline extraction of microsomes from EDEM1‐FLAG transfected cells with control or JB12‐HA to separate soluble and membrane‐anchored proteins from the ER. Representative ER‐soluble and membrane proteins, CRT and CNX, and the single transmembrane protein JB12 (endogenous and JB12‐HA) were fractionated into supernatants and pellets fractions, respectively (Figure 8d, bottom panel), indicating successful fractionation of microsomal proteins. Upon coexpression with the control vector, the transmembrane form of EDEM1‐FLAG, which migrated slower than the soluble fraction, was dominant (Figure 8d, lanes 2 and 3). However, coexpression of JB12‐HA decreased the ratio of transmembrane EDEM1‐FLAG (Figure 8d, lanes 5 and 6). This reduction in transmembrane EDEM1‐FLAG was consistent with the functional characteristics of JB12, which prefers ERAD client proteins bearing single‐ or multi‐spanning transmembrane region rather than soluble ERAD clients (Grove et al., 2011; Yamamoto et al., 2010). Collectively, our results suggest that multiple pathways of EDEM1 degradation are driven in and out of the ER, depending on the post‐translational modifications of EDEM1, such as mannose trimming of N‐glycans, disulfide bonds, and membrane topology.

## 3 DISCUSSION

### 3.1 EDEM1 degradation is in two ways, ERAD and autophagy

Initially, two degradation pathways of EDEM1, autophagy and the proteasome, were reported (Calì et al., 2008), and autophagy‐mediated EDEM1 degradation mechanisms, that is, segregation from the ER, colocalization with the autophagosome marker LC3, and ER‐phagy by the polyubiquitin receptor p62, have been proposed (Calì et al., 2008; Le Fourn et al., 2009; Park et al., 2014). In this study, we focused on an alternative EDEM1 degradation pathway, the proteasome, and found that EDEM1 turnover was facilitated by ERAD‐related factors through the ERAD pathway.

Aggregates of EDEM1‐FLAG, which appeared after cotreatment with MG132 and Clq, migrated more slowly than the soluble fraction (Figure 2a, lanes 7 and 8). Deglycosylation of EDEM1‐FLAG in both the supernatant and precipitation samples treated with MG132 and Clq resulted in a difference in band positions (Figure S1b), suggesting that the aggregation of EDEM1‐FLAG occurs more frequently in the transmembrane form by slow post‐translational signal sequence cleavage (Tamura et al., 2011) or unknown post‐translational modification than in the soluble form. As selective autophagy targets large protein aggregates (Gatica et al., 2018), our data suggest that autophagy selectively targets EDEM1‐FLAG aggregates caused by proteasome inhibition. Although the detailed pathway needs to be inspected, our results suggest that autophagy serves as a backup for the proteasome in the clearance of accumulated EDEM1.

### 3.2 EDEM1 as an ERAD substrate in various routes

The ubiquitination of EDEM1 has already been demonstrated by Park et al., who suggested that EDEM1 ubiquitination is required for recognition by p62, delivering polyubiquitinated proteins to autophagosomes through interaction with LC3 for subsequent autophagy degradation (Park et al., 2014). Here, we demonstrated that the deubiquitinase activity of YOD1, an ERAD‐related deubiquitinase associated with p97, is involved in EDEM1 clearance. YOD1 C160S, a catalytically inactive form (Ernst et al., 2009), exhibited an unfavorable effect on the degradation of EDEM1‐FLAG; increased detergent‐insoluble EDEM1‐FLAG, polyubiquitin‐positive accumulation of EDEM1‐FLAG was observed by indirect immunostaining, and increased ubiquitinated EDEM1‐FLAG (Figure 4). Ernst et al. proposed that YOD1 C160S stalled the dislocation of ERAD substrates due to a significant delay in deubiquitination (Ernst et al., 2009; Locke et al., 2014), suggesting that smooth deubiquitination by YOD1 is critical for EDEM1 degradation.

Several studies have reported that XTP3B negatively regulates ERAD through N‐linked glycan binding. XTP3B is a member of the SEL1L/Hrd1 complex that includes BiP (Hosokawa et al., 2008) and GRP94 (Christianson et al., 2008). The hypothesis that XTP3B negatively affects ERAD has been supported by studies using the widely used soluble ERAD model protein NHK; overexpression of XTP3B retards the ERAD of N‐glycosylated NHK (Iida et al., 2011) or non‐N‐glycosylated NHK (van der Goot et al., 2018), and siRNA to XTP3B accelerates the ERAD of NHK (Christianson et al., 2008; Fujimori et al., 2013; Zhong et al., 2015). XTP3B and OS9 deliver misfolded proteins to the SEL1L/Hrd1 complex through N‐glycans of SEL1L (van der Goot et al., 2018). Fujimori et al. showed that XTP3B recognizes Man9 N‐glycans on immature proteins and proposed that XTP3B protects these proteins from rash ERAD, thereby suppressing NHK degradation (Fujimori et al., 2013). In contrast, OS9 had a positive effect on the ERAD. OS9 enhances the ERAD of non‐glycosylated sonic hedgehog mutants in cooperation with EDEM2 (Tang et al., 2014), whereas OS9 promotes renal‐specific Na‐K‐2Cl co‐transporter degradation through ERAD, which is dependent on the client N‐glycan (Seaayfan et al., 2016). The KD of OS9 retards the ERAD of NHK or unassembled CD147 (an endogenous client of OS9), and a mutation in the MRH domain of OS9, which recognizes trimmed N‐glycans, negatively affects OS9‐dependent ERAD (Hosokawa et al., 2009; van der Goot et al., 2018). Interestingly, the downregulation of XTP3B by siRNA‐mediated knockdown or knockout cell lines moderately increased OS9 protein expression (Hattori et al., 2021; Manica et al., 2021; van der Goot et al., 2018; Zhong et al., 2015). We found that OS9 protein expression was downregulated by the co‐expression of XTP3B (Figure S10, lanes 1 to 3), which is consistent with previous reports and suggests that XTP3B can reduce OS9 protein expression. This reduction was partially inhibited by the proteasome inhibitor MG132 but not by mannose‐trimming or lysosome inhibitors (Figure S10, lanes 4 to 6). In this study, we found that EDEM1 was cleared by XTP3B‐mRFP overexpression in a KIF‐dependent manner, whereas OS9 overexpression did not affect EDEM1 turnover (Figure 6). Our results support the notion that the differential role of OS9 and XTP3B in ERAD.

### 3.3 The reason for short EDEM1 turnover for ER proteostasis

Proteostasis refers to a balanced state between protein production and degradation, and is executed in numerous situations and locations, such as the ER, cytoplasm, or whole body (Sala et al., 2017). Failure of ER proteostasis results in the accumulation of aberrant proteins and causes ER stress. Under ER stress, the ER protein balance is favored for ERAD and chaperone‐mediated refolding through the UPR. After alleviation of ER stress, the production of secretory and membrane proteins resumes. Simultaneously, the protein levels of EDEM1 and other ERAD‐enhancing factors must be lower than that of ER stress because overexpression of EDEM1 intercepts folding intermediates from the CNX folding cycle or the lag phase to ERAD, resulting in a decline in protein production (Molinari et al., 2003; Oda et al., 2003). Such a decrease in protein production by EDEM1 overexpression has also been observed for tyrosinase (Hagiwara et al., 2016; Marin et al., 2012). In contrast, the downregulation of EDEM1 expression affects the stability of WT ATF6 (Papaioannou et al., 2018), rod opsin (Kosmaoglou et al., 2009) and the hepatitis C virus envelope protein E2 (Saeed et al., 2011). Recently, we identified epidermal growth factor (EGF) receptor and thrombospondin‐1 as endogenous clients of EDEM1 (Miura et al., 2023). We also revealed an association between the EGF receptor and EDEM1‐FLAG and showed that EDEM1 promotes EGF receptor degradation through the ERAD pathway (Miura et al., 2023). In contrast, WT A1AT is not affected by transient expression of EDEM1 (Cormier et al., 2009; Hosokawa et al., 2006; Papaioannou et al., 2018) or stable EDEM1 expression in cell lines (Hosokawa et al., 2006). Collectively, these observations lead to the assumption that simple proteins like A1AT (three N‐glycans, no disulfide bonds, and 418 amino acids) easily reach the native state than others (for example, the EGF receptor contains one transmembrane region, 13 N‐glycans, and 25 disulfide bonds in 1210 amino acids). Under basal conditions, protein misfolding in the ER can be within a permissible range, and a certain amount of newly translated proteins is misfolded and degraded by the ERAD. Therefore, if UPR‐upregulated EDEM1 remains after restoration from ER stress, EDEM1 could cause excess degradation of de novo produced proteins, especially those with a complex structure, such as the EGF receptor. Therefore, the rapid turnover of EDEM1 may contribute to the protein maturation machinery after the end of ER stress.

ER‐resident chaperones are often turned slowly, whereas this process for ERAD‐related proteins is rapid. Representative ER chaperones or folding enhancement factors, such as BiP (>2 days) (Gülow et al., 2002), HSP47 (>24 h) (Nagata & Yamada, 1986), (>7 days) (Ohba et al., 1981), and GRP94 (>48 h) (Ostrovsky et al., 2010), appear to have a long half‐life. ERAD‐enhancing or modifying enzymes including EDEM1 (<4 h; Calì et al., 2008; Lamriben et al., 2018; Le Fourn et al., 2009), ER mannosidase I (0.5 h; Termine et al., 2009), overexpressed SEL1L (1 h; Iida et al., 2011), JB12 (6 h; Sopha et al., 2017), XBP1u (2 h; Yoshida et al., 2006), XBP1s (10–20 min; Liu et al., 2016), and ATF6 (2 h; Haze et al., 1999). We also confirmed that the turnover rate of ERAD‐related factors was relatively faster than that of ER‐resident chaperones and ERQC factors (Figure S11a). As far as we examined, transient expression of ERAD‐related factors used in this study did not exhibit a significant change in protein expression level and also ER stress confirmed by BiP (Figure S11b). Based on these insights, we speculated that ERAD‐enhancing proteins upregulated by the UPR are superfluous after ER stress is relieved. As such proteins undergo rapid turnover, unlike chaperones, the balance between protein production and degradation in the ER may return to an ordinary state after ER stress alleviation. Rapid turnover of EDEM1 has been proposed to tune EDEM1 levels when ER stress is alleviated (Merulla et al., 2013). In this study, we describe the degradation machinery of EDEM1 through several ERAD pathways and autophagy. A summary diagram from this study is shown in Figure S12. Although it is unclear which pathway is the principal or backup route, the resultant short half‐life of EDEM1 may contribute to the maintenance of lower cellular EDEM1 protein levels after diminishing ER stress to return to basal conditions.

## 4 EXPERIMENTAL PROCEDURES

### 4.1 Plasmids

A plasmid expressing the C‐terminal FLAG‐tagged human EDEM1, pCX4‐bsr‐EDEM1‐Flag, was constructed as previously described (Miura et al., 2023). Plasmids expressing Hrd1 wild‐type (WT)‐myc, Hrd1 C329S‐myc (Morito et al., 2008), SEL1L‐HA (Hosokawa & Wada, 2016), and OS9‐HA variant 2 (Hosokawa et al., 2009) were provided by Dr. Nobuko Hosokawa (Kyoto University, Japan). Plasmids expressing N‐terminal S‐tagged BAG6 (Minami et al., 2010) were kindly provided by Dr. Hiroyuki Kawahara (Tokyo Metropolitan University, Japan). Plasmids expressing mCherry‐ubiquitin (mCherry‐Ub) were a gift from Dr. Akira Kitamura (Hokkaido University, Japan). Plasmids expressing VIMP (Vcp/p97‐interacting membrane protein (VIMP) tagged with cfSGFP2 (cysteine‐free SGFP2) and the long isoform of XTP3B‐monomeric Red Fluorescent Protein (mRFP) were provided by Dr. Ikuo Wada (Fukushima Medical University, Japan). Plasmids expressing HA‐YOD1 WT, ERdj3‐myc, and JB12 WT‐HA constructed for this study were cloned by reverse transcription and PCR using specific primers listed in Table S1. Plasmid DNA sequence was confirmed using standard Sanger sequencing. Point mutations at C160S of HA‐YOD1, H53Q of ERdj3‐myc, and H138Q of JB12‐HA were introduced by standard inverse PCR with corresponding mutagenic primers.

### 4.2 Antibodies and chemicals

MG132 was obtained from the Peptide Institute Inc. (Osaka, Japan). Triton X‐100 (TX100), E64 (an antipain agent), and N‐(2‐hydroxy ethyl)‐piperazine ethane sulfonic acid (HEPES) were purchased from Nacalai Tesque (Kyoto, Japan). Leupeptin, pepstatin, CHX and anti‐FLAG agarose beads were purchased from Sigma‐Aldrich (St. Louis, MO, USA). WM was purchased from Calbiochem (Darmstadt, Germany). Clq, KIF, and Protein N‐glycanase F (PNGaseF) were purchased from FUJIFILM Wako Pure Chemical Corporation (Osaka, Japan), Enzo Life Sciences (Farmingdale, NY, USA), and New England Biolabs (Ipswich, MA, USA). The antibodies used in this study are listed in Table S2, along with their dilutions in each experiment.

### 4.3 Cell culture, transfection, drug treatment, and western blotting

HEK293 EBNA and HeLa cells were kindly provided by Dr. Ikuo Wada. Cells were grown at 37°C in 5% CO_2_ with humidity and were maintained in Dulbecco's modified Eagle's medium (DMEM; Invitrogen, Carlsbad, CA, USA) containing 10% fetal bovine serum (FBS; Invitrogen), 1 mM L‐glutamine (Nacalai tesque), and 1% penicillin/streptomycin (Nacalai Tesque). Cells were authenticated using a Universal Mycoplasma detection Kit (e‐MycoTM, iNtRON, South Korea).

To introduce protein expression plasmids into the cells, 293 EBNA and HeLa cells were treated with polyethyleneimine (Polysciences Inc., Warrington, PA, USA) and HilyMax (DOJINDO Laboratories, Kumamoto, Japan), respectively, according to the manufacturer's instructions. The pcDNA 3.1 vector (Invitrogen) was used as the control vector.

Before cell lysis, the cells were washed once with PBS and incubated on ice with PBS containing 10 mM *N*‐ethylmeleimide (Kanto Chemical Co. Inc., Tokyo, Japan) for 10 min to modify free thiol groups and prevent further modifications. Then, cells were solubilized in the lysis buffer containing 1% TX100, 20 mM HEPES, pH = 7.5, 150 mM NaCl, and protease inhibitor mix (5 μg/mL E64, antipain, leupeptin, and pepstatin). The lysate was centrifuged at 4°C, 18,000 × *g* for 10 min to obtain soluble (supernatant) and insoluble (precipitates) fractions. After the addition of sodium dodecyl‐sulfate polyacrylamide gel electrophoresis (SDS‐PAGE) sample buffer, the proteins in the supernatant were denatured at 90°C for 5 min. The precipitates were solubilized with the same amount of lysis buffer containing the SDS‐PAGE sample buffer by pipetting, sonication, and heat denaturation, as described above. Denatured proteins were separated by reducing (denatured with 0.1 M dithiothreitol) SDS‐PAGE and then transferred to a polyvinylidene difluoride (PVDF) membrane (Immobilon; Millipore, Burlington, MA, USA). After blocking with 5% skim milk in 50 mM Tris–HCl (pH 7.5) containing 100 mM NaCl and 0.05% Tween‐20 at 2°C overnight, the membranes were incubated with primary antibodies at 25°C for 1 h. After washing with Tris buffer without skim milk, the membranes were treated with horseradish peroxidase (HRP)‐conjugated goat anti‐rabbit or mouse secondary antibodies (Sigma‐Aldrich) at 25°C for 30 min. Protein bands of interest were detected using a chemiluminescence reagent (luminol, *p*‐coumaric acid, and hydrogen peroxide) and a ChemiDoc system (Bio‐Rad, Hercules, CA, USA). Densitometric analysis was performed using the ImageJ software v1.53 (National Institute of Health, Bethesda, MD, USA). To analyze protein turnover, transfected 293 EBNA cells were treated with above media containing 10 μg/mL CHX for the indicated times. In the drug treatment assay, culture media were replaced 32 h after transfection with drug‐containing medium and then incubated for 16 h. For deglycosylation analysis, denatured cell lysates were treated with PNGase F, according to the manufacturer's instructions. The expression of proteins of interest in western blotting experiment was normalized to that of αTubulin as a cellular loading control. In all western blotting images, proteins of interest and molecular weights of marker proteins are shown on the left and right sides of the blot, respectively. The lane numbers are indicated at the bottom. Data are shown as the mean of at least three independent experiments and were compared using a two‐tailed Student's t‐test (**p < 0.01, *p < 0.05, N.S., not significant compared to the control). Error bars represent the standard deviation between experiments.

### 4.4 Immunoprecipitation

Cell lysates obtained after transfection of 293 EBNA cells were separated into two fractions: input (total proteins) and lysate for the pull‐down assay. For immunoisolation of FLAG‐tagged proteins, the cell lysate was incubated with anti‐FLAG M2 agarose beads (Sigma‐Aldrich) at 4°C for 1 h with gentle rotation, followed by centrifugation at 3000 rpm for 10 min at 4°C. The precipitated beads were washed three times with 50 mM Tris–HCl (pH 7.5) containing 0.1% 3‐[(3‐cholamidopropyl) dimethylammonio] propanesulfonate (CHAPS) and 150 mM NaCl. EDEM1‐FLAG and associating proteins were eluted form the beads by incubation with 500 μM FLAG peptide (Sigma‐Aldrich) in 50 mM Tris–HCl (pH 7.5) containing 150 mM NaCl at 25°C for 15 min with gentle rotation. Extracted proteins were denatured and analyzed by western blotting as described above. For immunoisolation of HA‐tagged JB12, cell lysates were incubated with mouse anti‐HA (MBL Japan, Tokyo, Japan) at 4°C for 1 h. The cell lysates were then incubated with protein‐A Sepharose (GE Healthcare, Chicago, Illinois, USA) at 4°C for 1 h with gentle rotation, followed by washing as described above. SDS sample buffer was added to the antibody complex, which was then denatured at 90°C for 5 min.

### 4.5 Indirect immunostaining

To visualize the cellular location of the proteins of interest, indirect immunostaining with fluorescently labeled secondary antibodies and confocal microscopy were performed as previously described (Tamura et al., 2013). Transfected HeLa cells grown on coverslips in a 35 mm dish were washed with PBS and fixed with 4% paraformaldehyde in PBS (FUJIFILM Wako) at 25°C for 10 min. Plasma membrane was permeabilized by treating with 0.1% of TX100 in immunostaining buffer (PBS containing 5% of glycerol and 1% of Goat serum) at 2°C for 1 min, then cells were blocked in above immunostaining buffer at 25°C for 5 min. The cells were then incubated with primary and secondary antibodies at 25°C for 20 min. After staining with DAPI (FUJIFILM Wako) at 25°C for 5 min, the coverslips were rinsed with distilled water and mounted face‐down on glass slides using Mowiol (Sigma‐Aldrich). Images were taken by a confocal laser microscope with an oil immersion objective under a magnification of 63× (LSM 780, Carl Zeiss, Inc., Oberkochen, Germany).

### 4.6 Alkaline extraction of microsomes

Soluble and membrane‐anchored proteins were separated using alkaline extraction methods based on a previous report (Tamura et al., 2011). Transfected 293 EBNA cells were washed with PBS and PBS containing 10 mM N‐ethylmaleimide as described above. Cells were suspended in homogenization buffer (20 mM HEPES, pH 7.5, 5 mM KCl, 120 mM NaCl, 1 mM EDTA, 0.2 M sucrose, and above protease inhibitor mix) and passed through 22G micro syringe 20 times. Cell lysates were centrifuged at 1000 *g*, 4°C for 10 min and the post‐nuclear supernatant was centrifuged at 36,000 rpm in Himac rotor (S55A2) for 10 min to isolate the microsomes. The pellet was suspended in PBS, supplemented with freshly prepared 0.1 M Na_2_CO_3_ (pH = 11.5) then incubated for 30 min on ice. Samples were divided into two fractions (total membrane and further step) and separated into soluble and pellet fractions by ultracentrifugation at 52,000 rpm in a Himac rotor (S55A2) for 15 min with a 0.3 M sucrose cushion in PBS. Proteins in the total membrane and supernatant were precipitated with trichloroacetic acid, washed with acetone, and denatured using SDS‐PAGE sample buffer. The pellet fraction was directly lysed and denatured using SDS‐PAGE sample buffer.

## Supporting information


**Data S1:** Supporting Information.
