# Supplementary material for: Turnover of EDEM1, an ERAD‐enhancing factor, is mediated by multiple degradation routes
Source: Genes Cells. 2024 Apr 29;29(6):486–502. doi: 10.1111/gtc.13117 (PMC11163939; doi:10.1111/gtc.13117)
Supplement: Supplementary file 1 — Data S1: Supporting Information. [file GTC-29-486-s001.pdf]

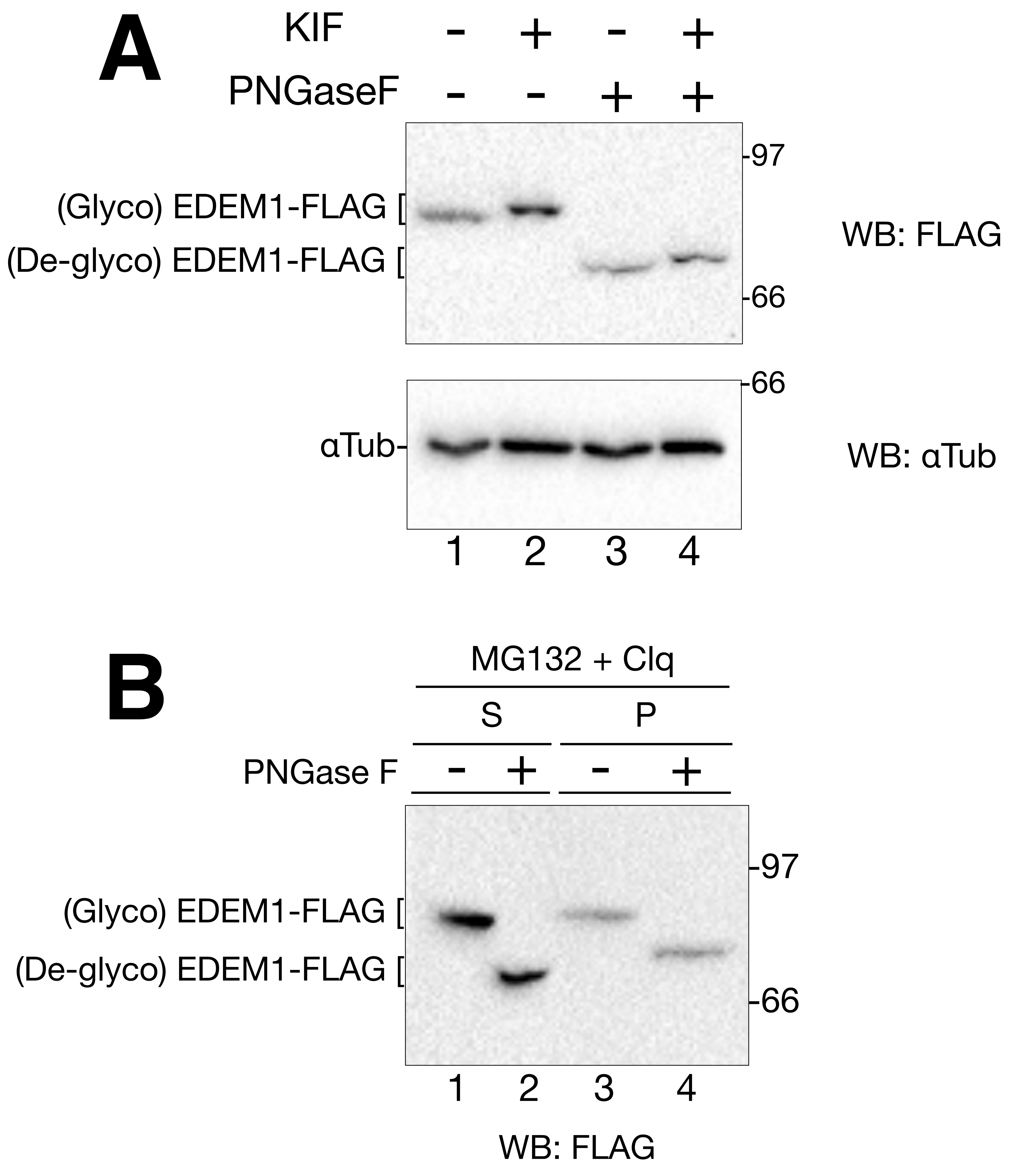

Suuplementary Figure 1
Biochemical analysis of EDEM1-FLAG by PNGaseF

(A) Protein samples of the supernatant obtained similar to Fig. 1C were subjected to mock-treated (lanes 1 and 2) or PNGaseF-treated (lanes 3 and 4). After reducing SDS-PAGE, proteins of interest were visualized using indicated antibodies. Of note, samples in lanes 2 and 4 for immunoblotting using anti-FLAG were loaded after 1:4 dilution to be distinguishable.

(B) S Samples (used in Fig.2A, lane 7) and P samples (used in Fig.2A, lane 8) were mock treated (-) or digested with PNGase F (+) to remove N-linked glycans of EDEM1-FLAG. Samples were resolved by reducing SDS-PAGE and immunoblotted using anti-FLAG antibody. Glyco and De-glyco EDEM1-FLAG are indicated at the left side of the blot.

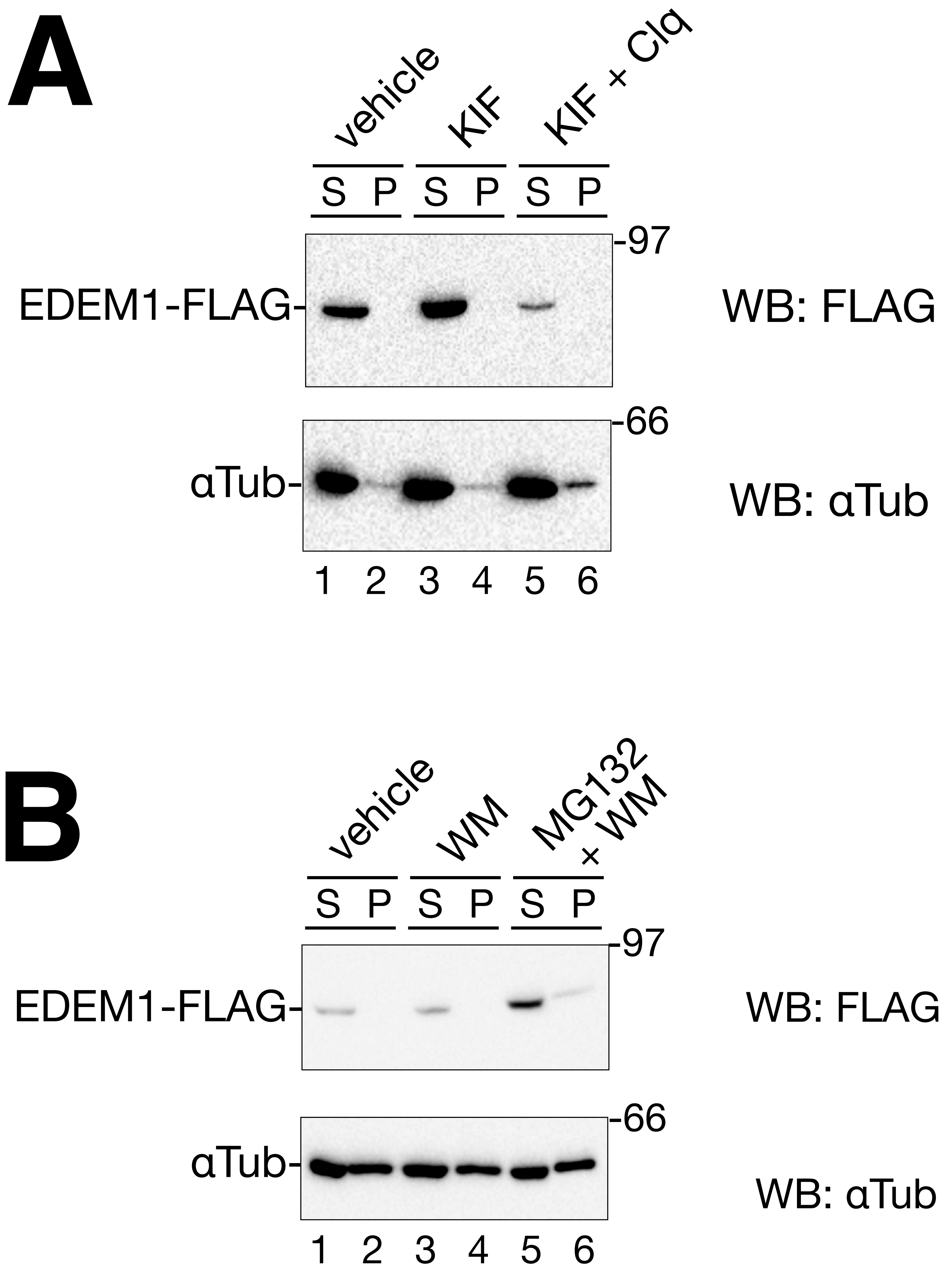

Suuplementary Figure 2
Analysis of the detergent-insoluble EDEM1-FLAG in the presence of KIF or WM

(A) 293 EBNA cells transfected with EDEM1-FLAG were treated with the indicated drug (150 μM KIF or KIF and 50 μM Clq) for 16 hr and cell lysates were separated into detergent-soluble (S) and insoluble fractions (P). Samples were resolved in reducing SDS-PAGE and immunoblotted using anti-FLAG and anti-αTubulin antibodies.

(B) 293 EBNA cells were transfected with EDEM1-FLAG for 48 hr and treated with the indicated drug (10 nM wortmannin (WM) or WM and 1 μM MG132) for the last 16 hr. Cell lysates were obtained and separated into detergent-soluble (S) and -insoluble fractions (P). Samples were resolved in reducing SDS-PAGE and immunoblotted using anti-FLAG and anti-αTubulin antibodies.

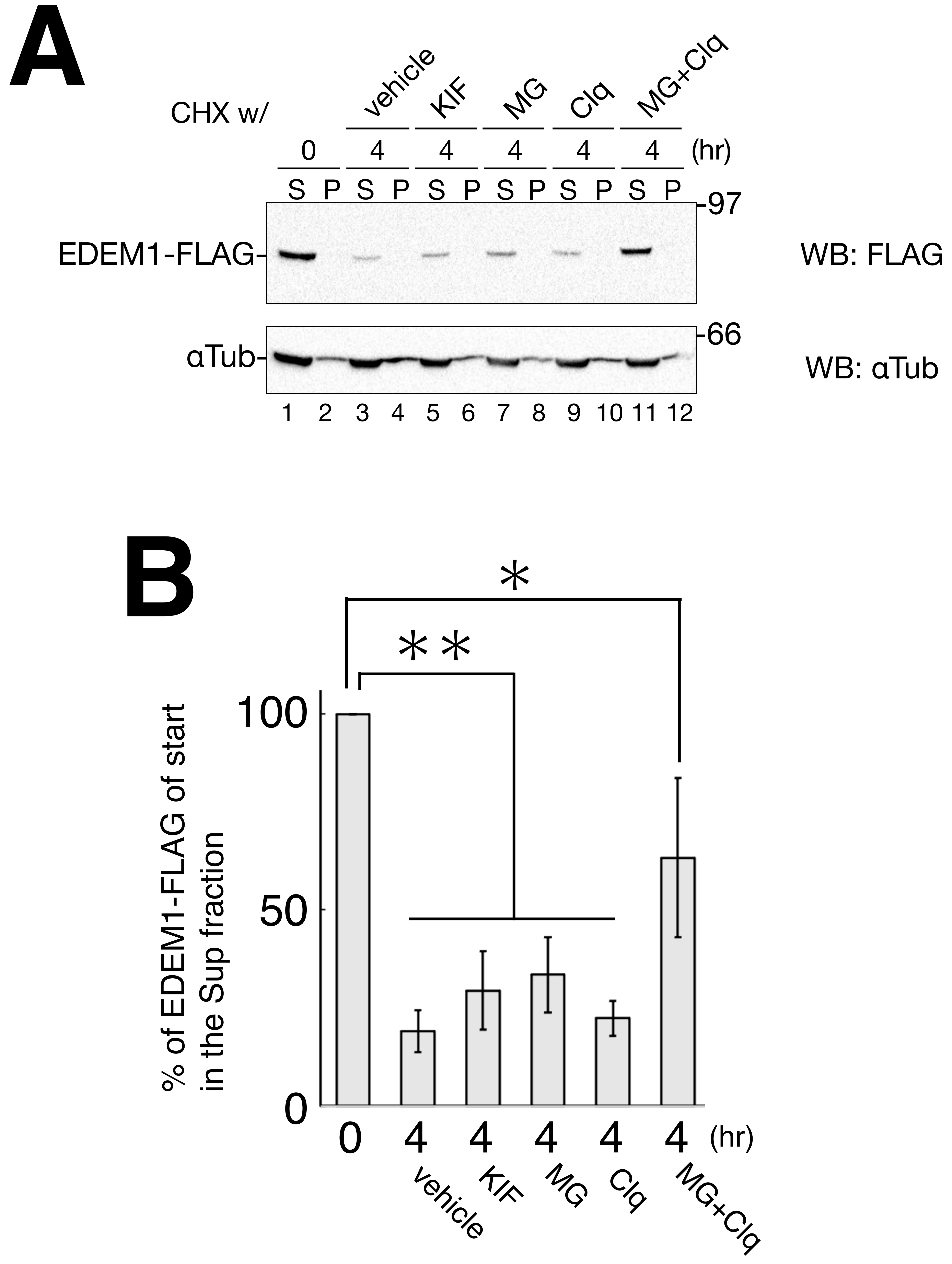

Supplementary Figure 3
Examination of EDEM1-FLAG turnover by CHX-chase

(A) 293 EBNA cells were transfected with EDEM1-FLAG for 48 hr then treated with 10 μg/mL CHX for 0 or 4 hr with or without indicated drugs (the same concentration as Fig. 1). αTubulin was used as the loading control. Western blotting was performed with the indicated antibodies.

(B) EDEM1-FLAG of odd number lanes of A were quantified and plotted by the three independent experiments.

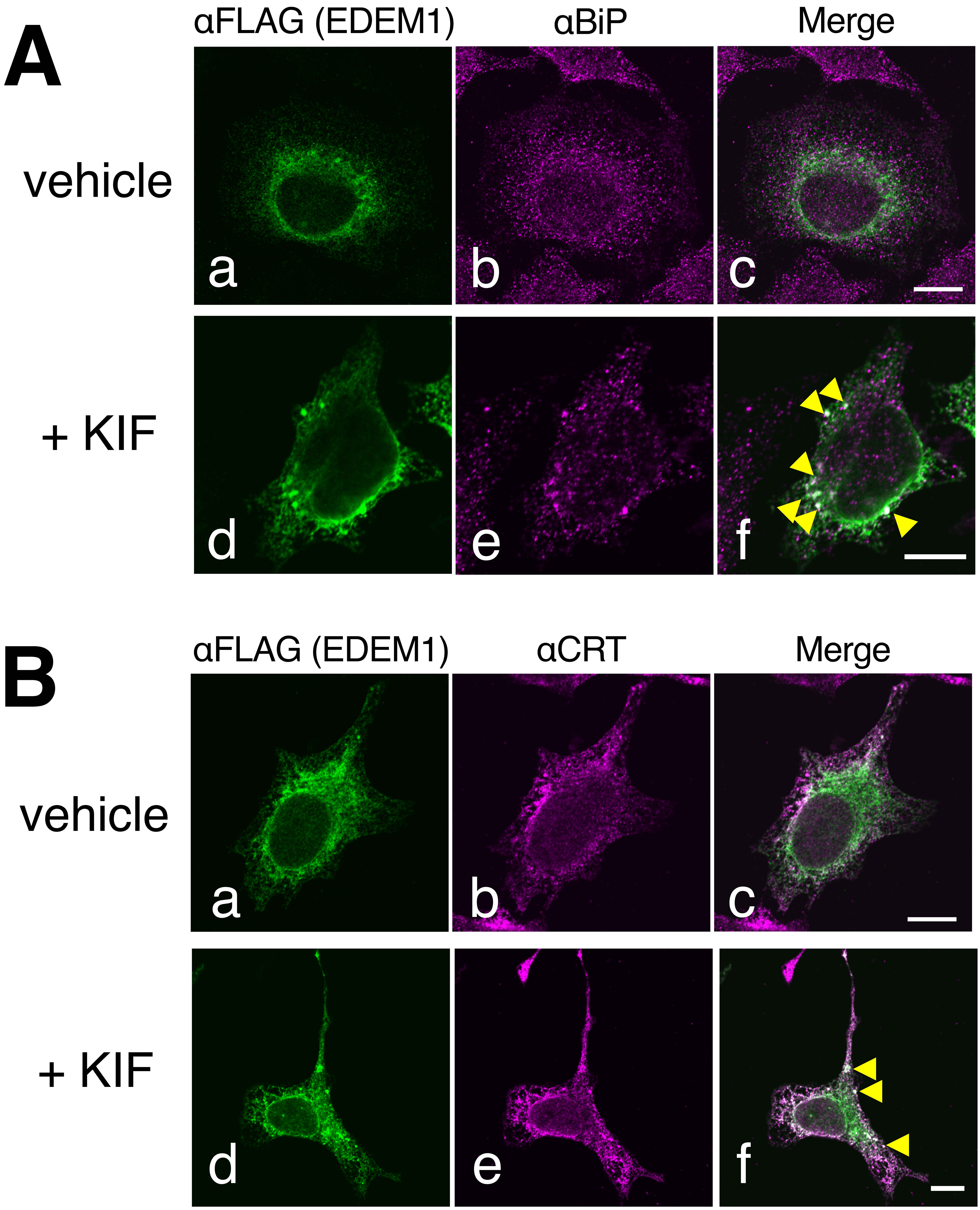

Suuplementary Figure 4
Cellular location of EDEM1-FLAG, BiP, and CRT under ERAD inhibition.

Transfected HeLa cells were treated with KIF as in Figure 3A and indirect immunofluorescence was performed using anti-FLAG (Green, 488, a and d) and anti-BiP (part A, magenta, 594, b and e) or anti-CRT (part B, magenta, 594, b and e). Merged images (c, f, and i) are presented with scale bars (10 μm). Part of colocalization is shown by yellow triangles.

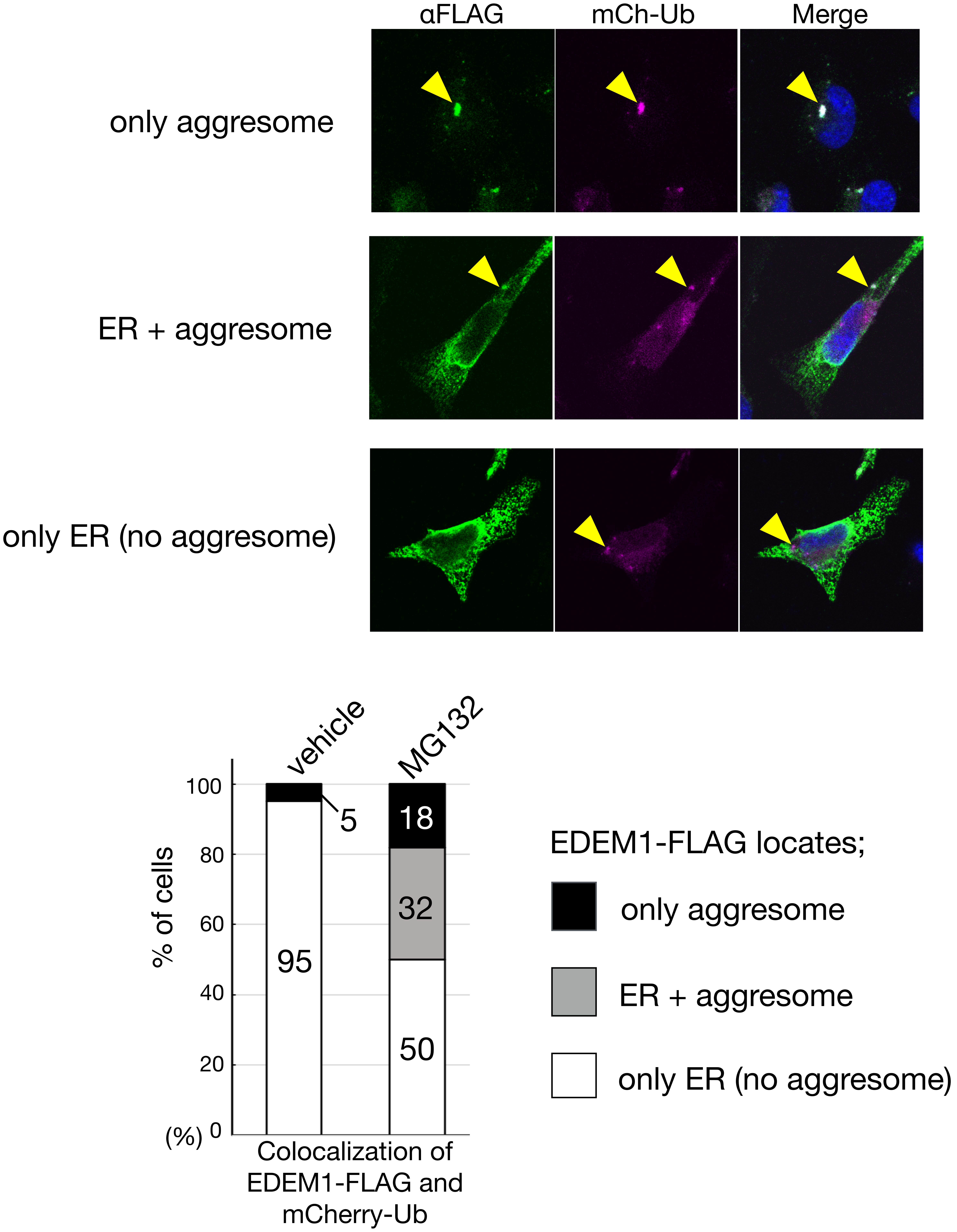

Suuplementary Figure 5
Quantification of cells that exhibit colocalization of EDEM1-FLAG and mCherry-Ub.

HeLa cells were co-transfected with EDEM1-FLAG and mCherry-Ub on the coverslip for 48 hr with vehicle or MG132 for the last 16 hr. After fixation with 4% PFA and permeabilization, indirect immunofluorescence was performed using anti-FLAG. Cells that co-express EDEM1-FLAG and mCherry-Ub were counted as total cell numbers. EDEM1-FLAG expression pattern was classified: cells that EDEM1-FLAG was only found at the aggresome and colocalized with mCherry-Ub are “only aggresome” as a black bar, cells that EDEM1-FLAG was located at both the ER and the aggresome are “ER+aggresome” as a dark grey bar, and cells that EDEM1-FLAG was found only at the ER are “only ER” as a white bar. Total cell numbers are 63 (vehicle) and 50 (MG132), respectively. The ratio of cells to the total number is shown.

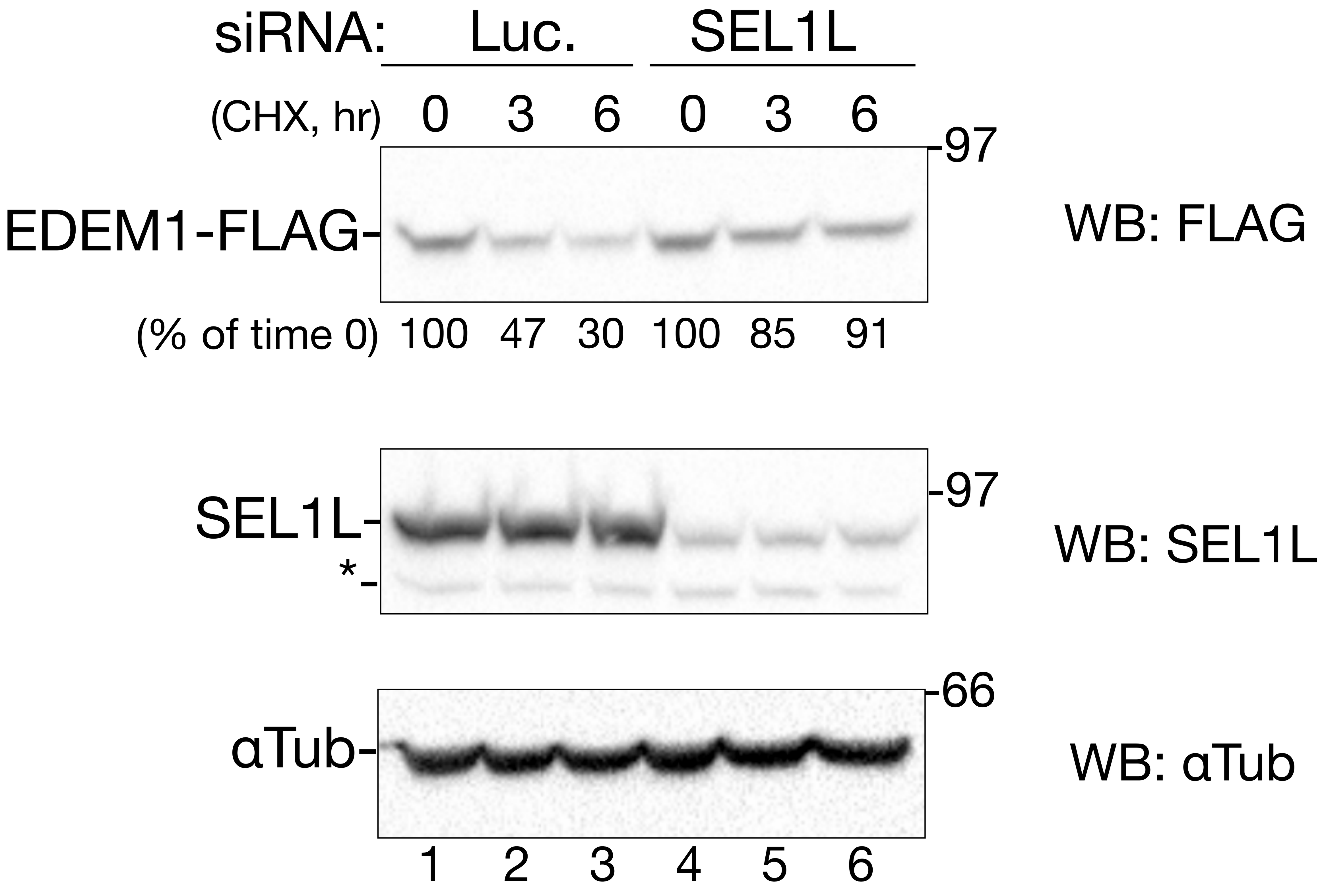

Suuplementary Figure 6
SEL1L knockdown slows EDEM1-FLAG degradation.

293 EBNA cells were treated with siRNA for 72 hr using control (Luciferase, Luc.) or SEL1L siRNA oligos. Cells were transfected with EDEM1-FLAG within the last 48 hr of siRNA. CHX-treatment of cells was conducted and after cell lysis, detergent-soluble fractions were resolved in reducing SDS-PAGE. Western blotting using indicated antibodies was performed. An asterisk in the SEL1L blot indicates the cellular nonspecific protein's band. Values of EDEM1-FLAG are normalized by that of αTubulin and set the time of each zero point as 100. RNA oligo for SEL1L (SEL1L-1, Hosokawa N et al, 2008) is kindly gifted from Dr. Nobuko Hosokawa (Kyoto University, Japan).

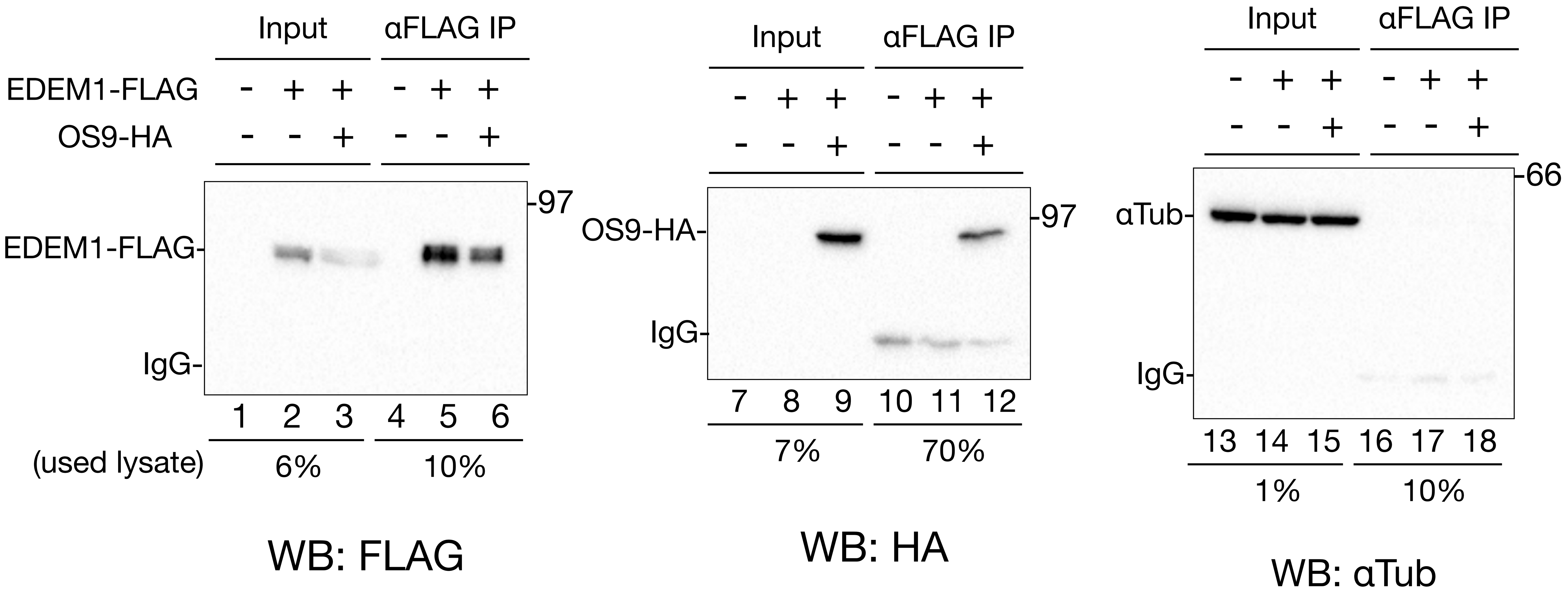

Suuplementary Figure 7
Physical Interaction of EDEM1-FLAG and OS9-HA

293 EBNA cells were transfected with the control vector, EDEM1-FLAG or EDEM1-FLAG and OS9-HA for 48 hr. Cells were lysed and detergent-soluble fraction were aliquoted for input or following immunoprecipitation (percentages for each experiments are indicated). Immunoisolation of EDEM1-FLAG using anti-FLAG agarose beads was operated. Samples were resolved in reducing SDS-PAGE and immunoblotted using with indicated antibodies.

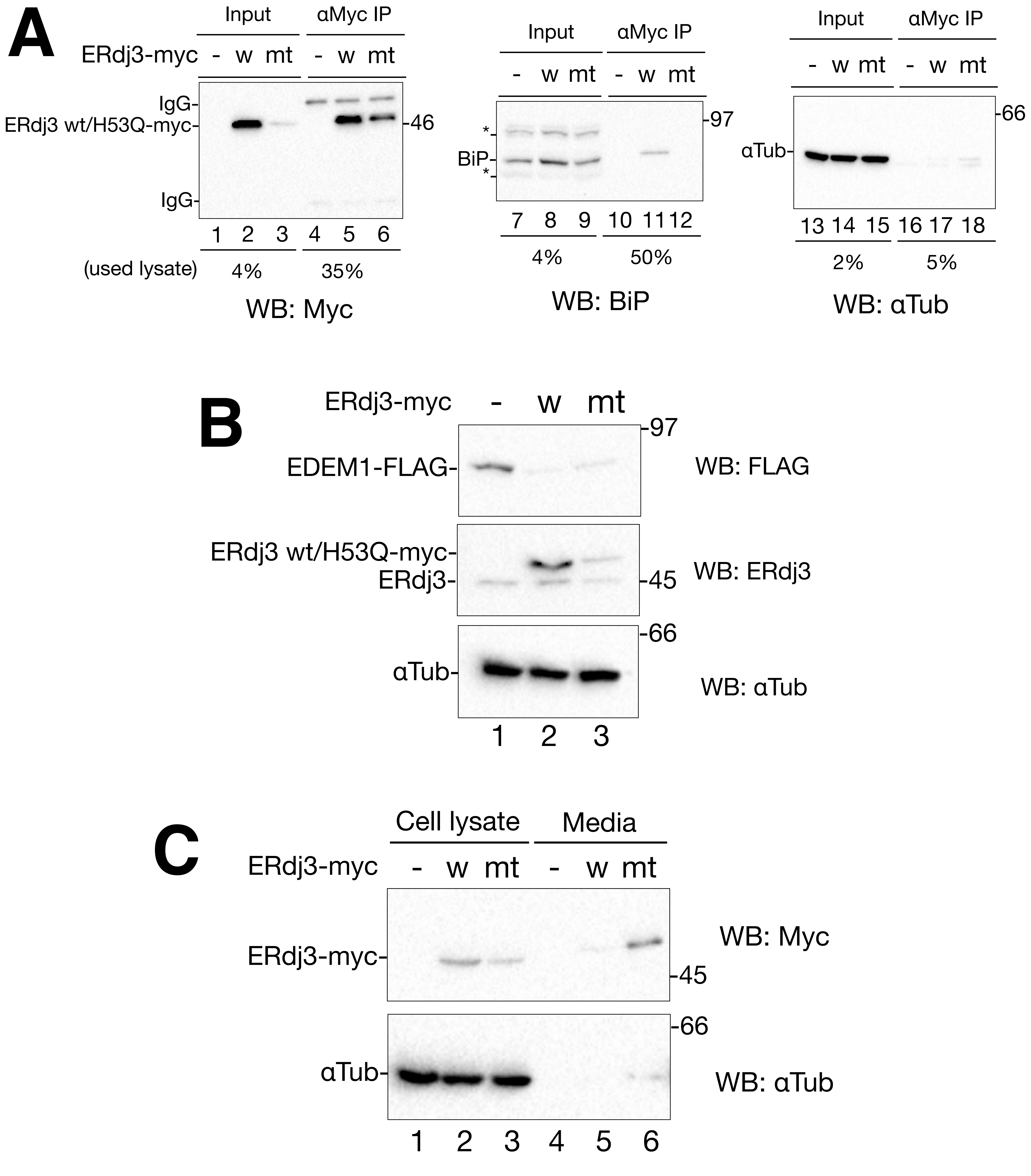

Suuplementary Figure 8
Wild type and J domain mutant of ERdj3 is not associated with EDEM1-FLAG degradation.

(A) 293 EBNA cells were transfected with control vector, ERdj3 wt-myc, or ERdj3 H53Q-myc for 48 hr. Cells were lysed and detergent-soluble fraction were aliquoted for input or following anti-Myc immunoprecipitation (percentage for each experiments are indicated). Immunoprecipitation using anti-Myc and protein-A beads was operated. Samples were resolved in reducing SDS-PAGE and immunoblotted using indicated antibodies.
(B) 293 EBNA cells were cotransfected with EDEM1-FLAG and control vector (lane 1), ERdj3 wt myc (lane 2), or ERdj3 H53Q-myc (lane 3) for 48 hr. Detergent-soluble cell lysates were resolved in reducing SDS-PAGE and immunoblotted using anti-FLAG, anti-ERdj3, and anti-αTubulin antibodies.
(C) 293 EBNA cells were transfected as (A) except for the last 16 hr cultured with serum-free media, OPTI-MEM (Moleclular probes, Eugene, OR, USA). After removing cells in media by centrifugation (1000 *g*, 4°C for 3 min), proteins secreted into the media were collected by a general methanol-chloroform precipitation method. Proteins in cell lysates and the media were resolved in reducing SDS-PAGE and immunoblotted using indicated antibodies.

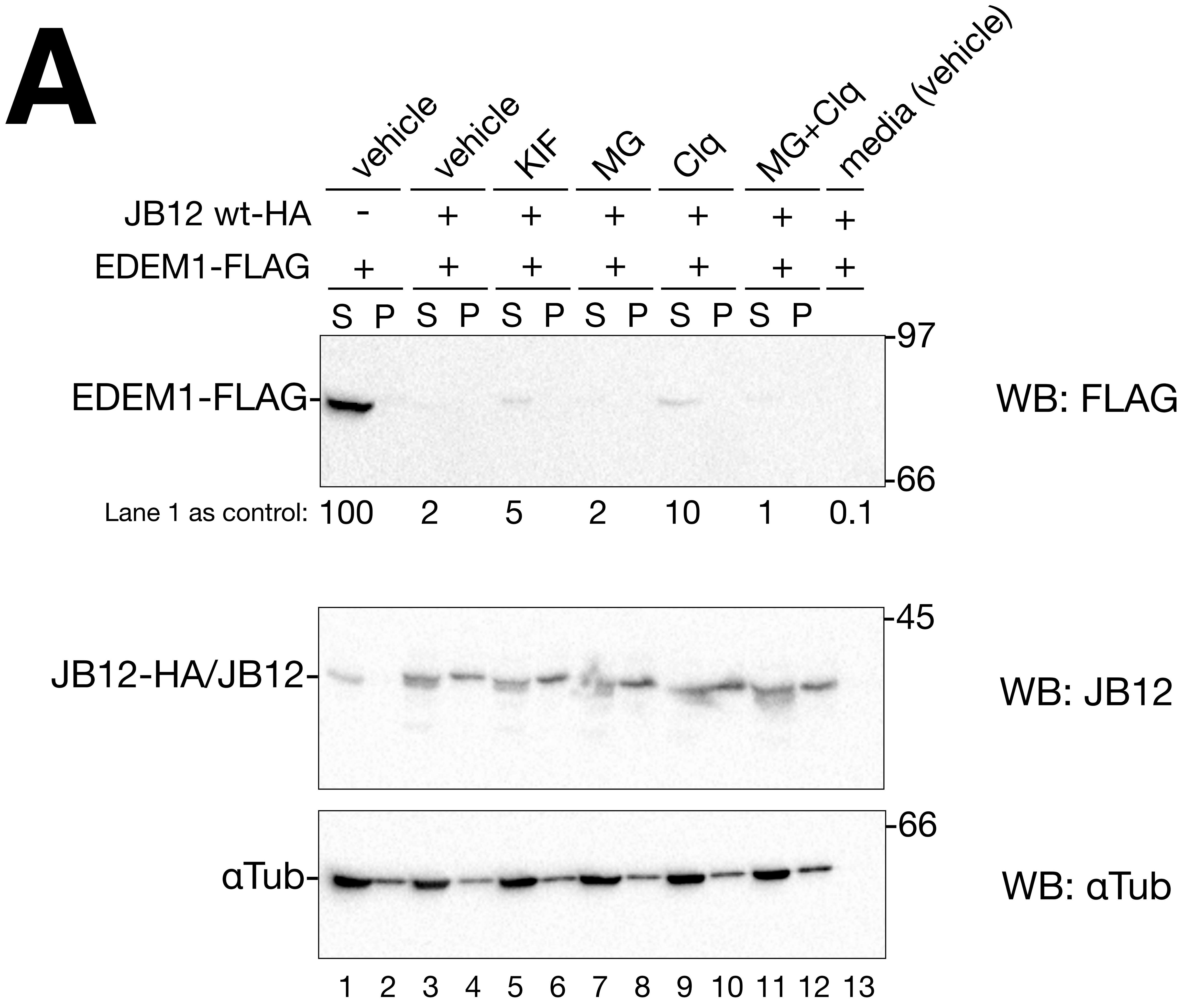

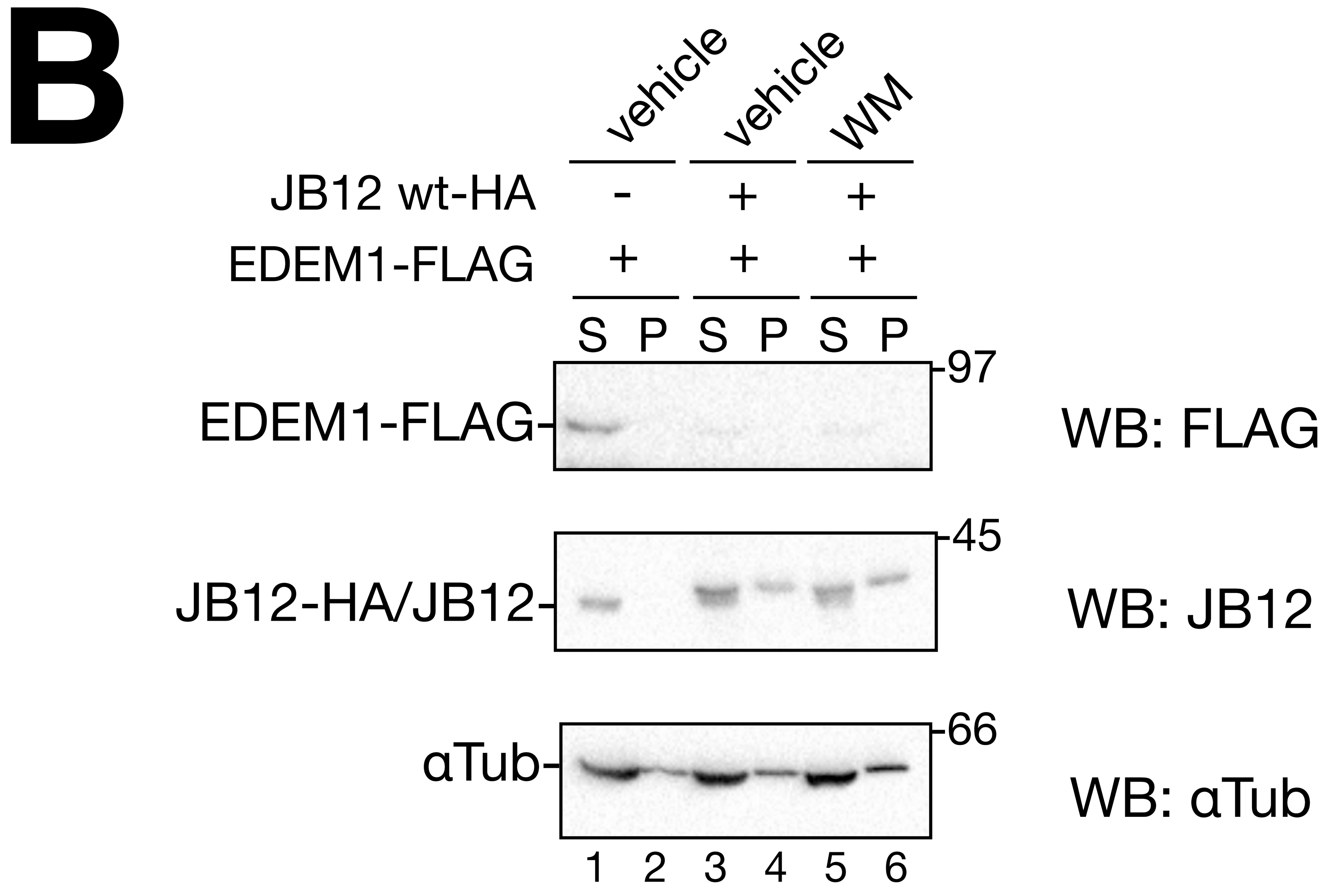

Suuplementary Figure 9
Effect of various drugs and the H128Q mutation on EDEM1-FLAG degradation enhanced by JB12.

(A) 293 EBNA cells were transfected with EDEM1-FLAG and empty vector or JB12-HA with or without indicated drugs (150 μM KIF, 1 μM MG132, 50 μM Clq, or MG132 and Clq) for 16 h. Cell lysates were separated into detergent-soluble (S) and -insoluble fractions (P). Proteins in the media (lanes 13, cultured with OPTI-MEM for the last 16 hr) were precipitated using standard methanol/chloroform methods. Samples were resolved by reducing SDS-PAGE and immunoblotted using indicated antibodies.
(B) Protein samples were prepared and subjected as C except for using 10 nM WM.

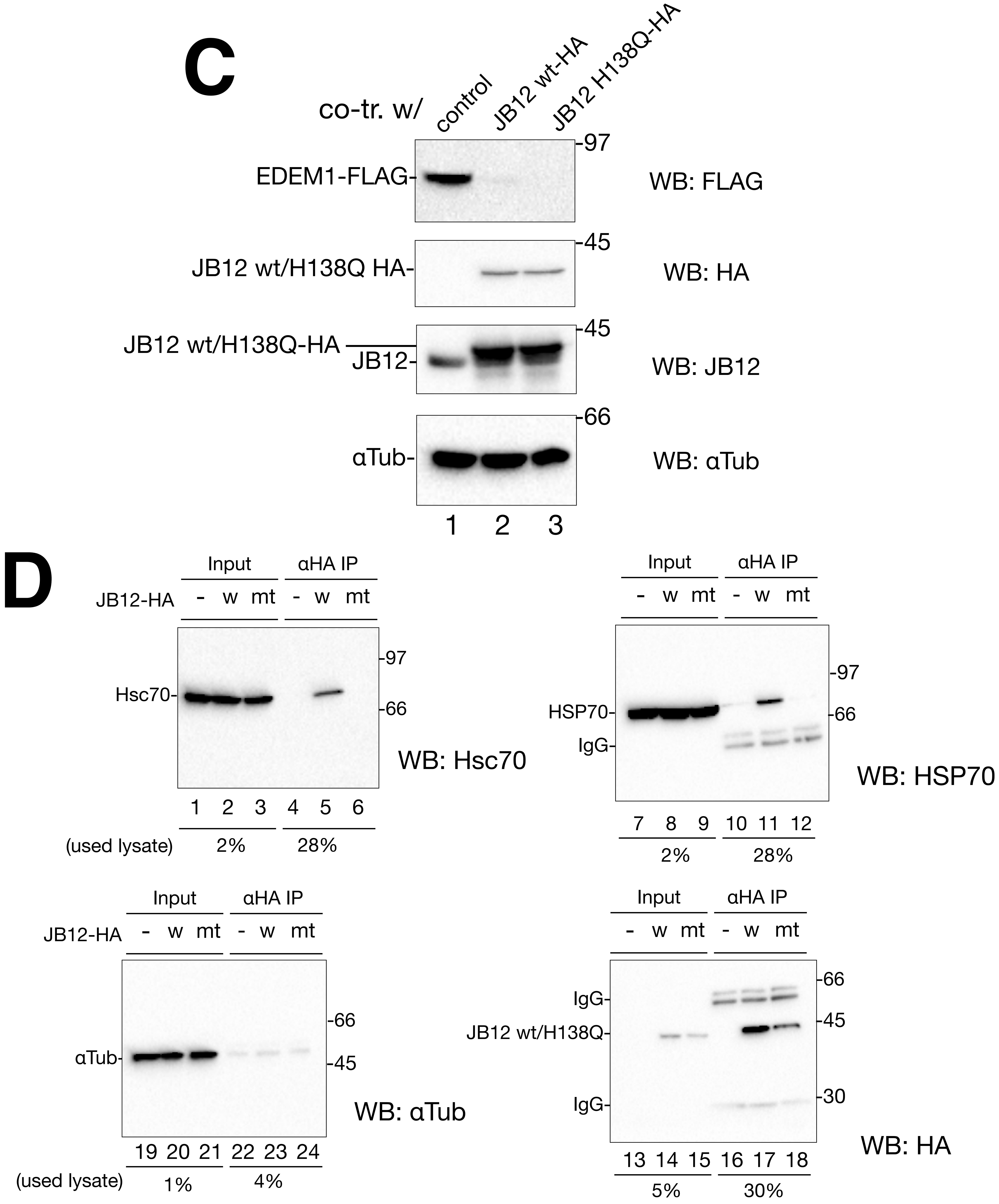

Supplementary Figure 9 continued

(C) 293 EBNA cells were cotransfected with EDEM1-FLAG and control vector (lane 1), JB12 wt-HA (lane 2), or JB12 H138Q-HA (lane 3), respectively for 48 hr. Detergent-soluble cell lysates were resolved in reducing SDS-PAGE and immunoblotted using indicated antibodies.
(D) 293 EBNA cells were transfected with the control vector, JB12 wt-HA (w), or JB12 H138Q-HA (mt) for 48 hr. Cells were lysed and detergent-soluble fractions were aliquoted for input or following anti-HA IP (percentages for each experiment are indicated). Immunoprecipitation using anti-HA and protein-A beads was operated. Samples were resolved by reducing SDS-PAGE and immunoblotted using indicated antibodies.

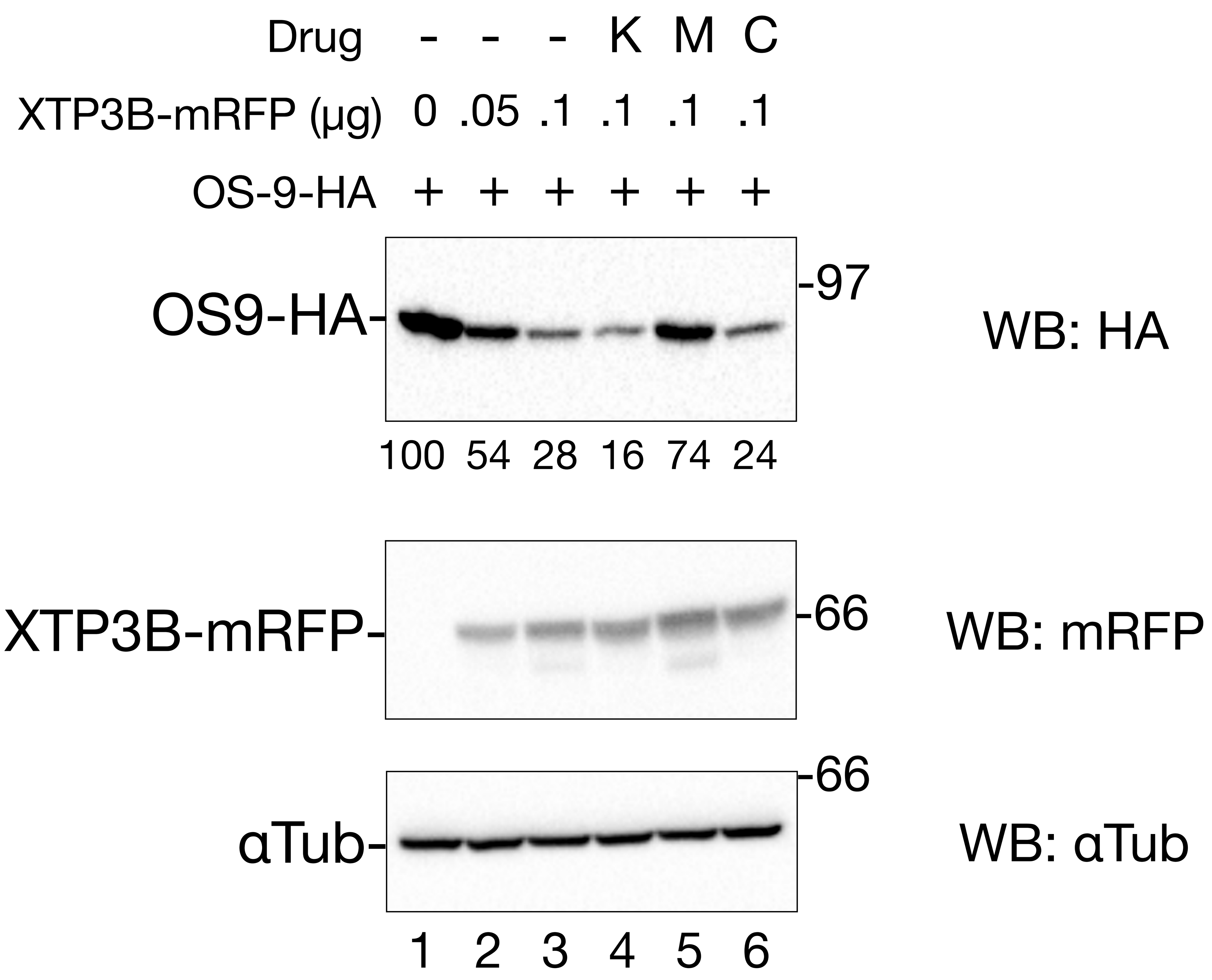

Suuplementary Figure 10
Down-regulation of OS9 by XTP3B is recovered by the proteasome inhibition

293 EBNA cells were transfected with OS9-HA and 0-0.1 μg of XTP3B-mRFP for 48 hr with indicated drug for the last 16 hr: K, 150 μM KIF; M, 1 μM MG132; C, 50 μM Clq, respectively. Detergent-soluble cell lysates were resolved in reducing SDS-PAGE. Immunoblotting using anti-HA, anti-mRFP and anti-αTubulin antibodies was perfomed. Expression level of OS9-HA (normalized with αTub) was indicated at the bottom of OS9-HA blot (value of lane 1 was set as 100 and the ratio to lane 1 was shown for each sample).

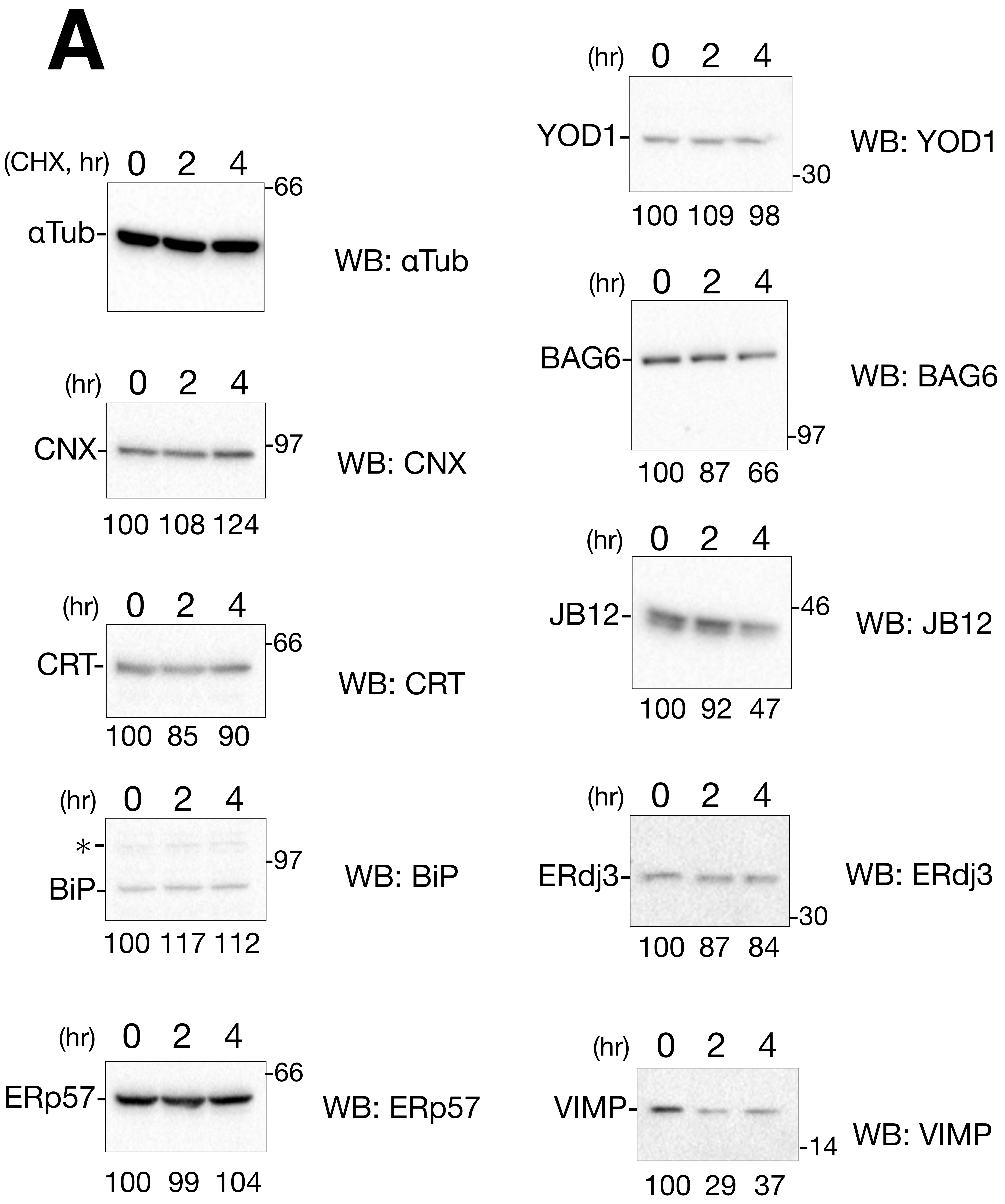

Suuplementary Fig. 11.
Protein expression analysis of ER-resident chaperones and ERAD-factors

(A) Samples used in Figure 1A were immunoblotted using indicated antibodies. The expression level of proteins of interest was normalized with αTub and the value was indicated at the bottom of each blot (the value of CHX-chase 0 hr was set as 100).

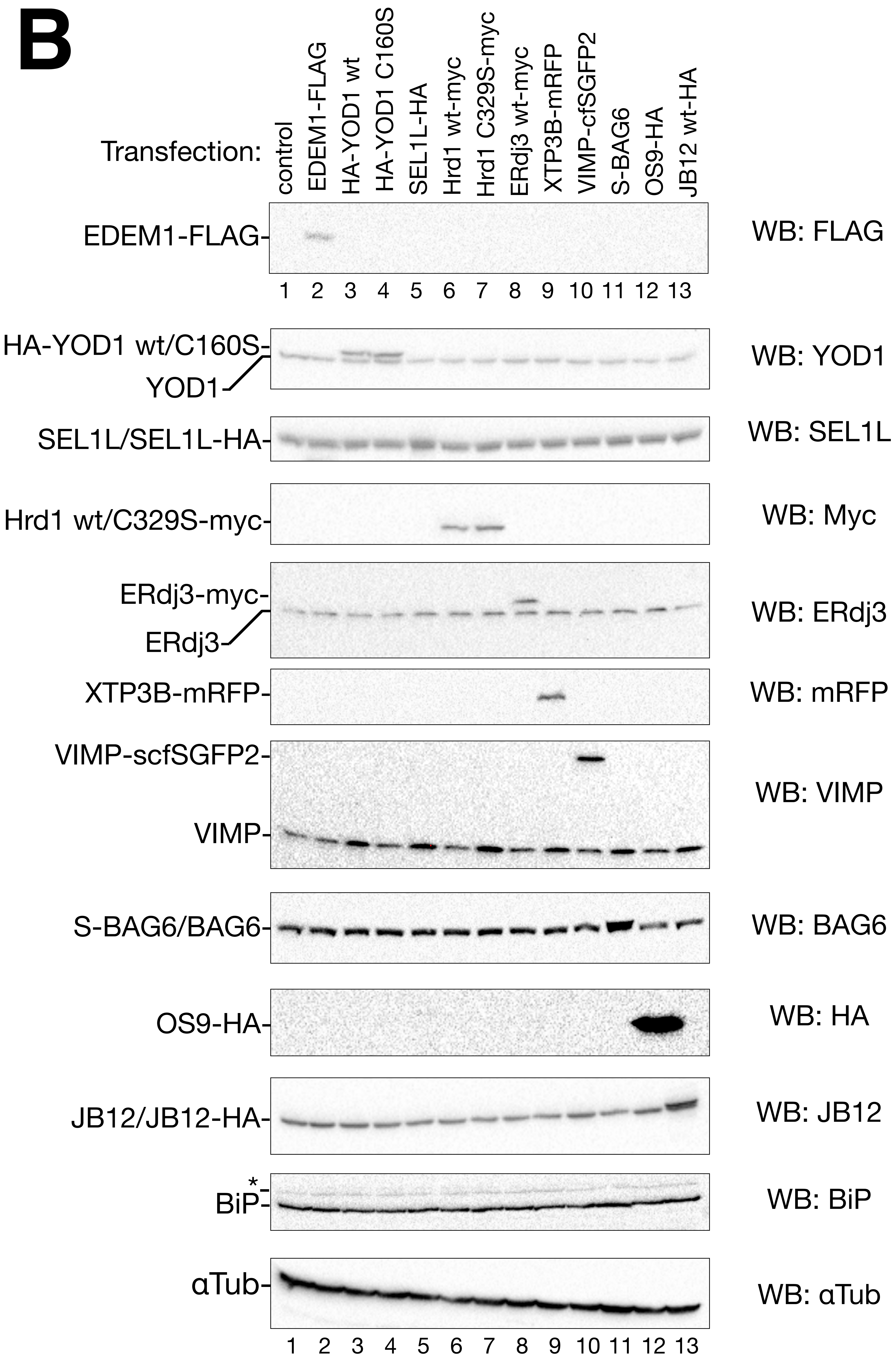

Suuplementary Figure 11 continued

(B) 293 EBNA cells were transfected with indicated vectors for 48 hr. Obtained samples prepared as described in Materials and Methods were resolved and immunoblotted using indicated antibodies.

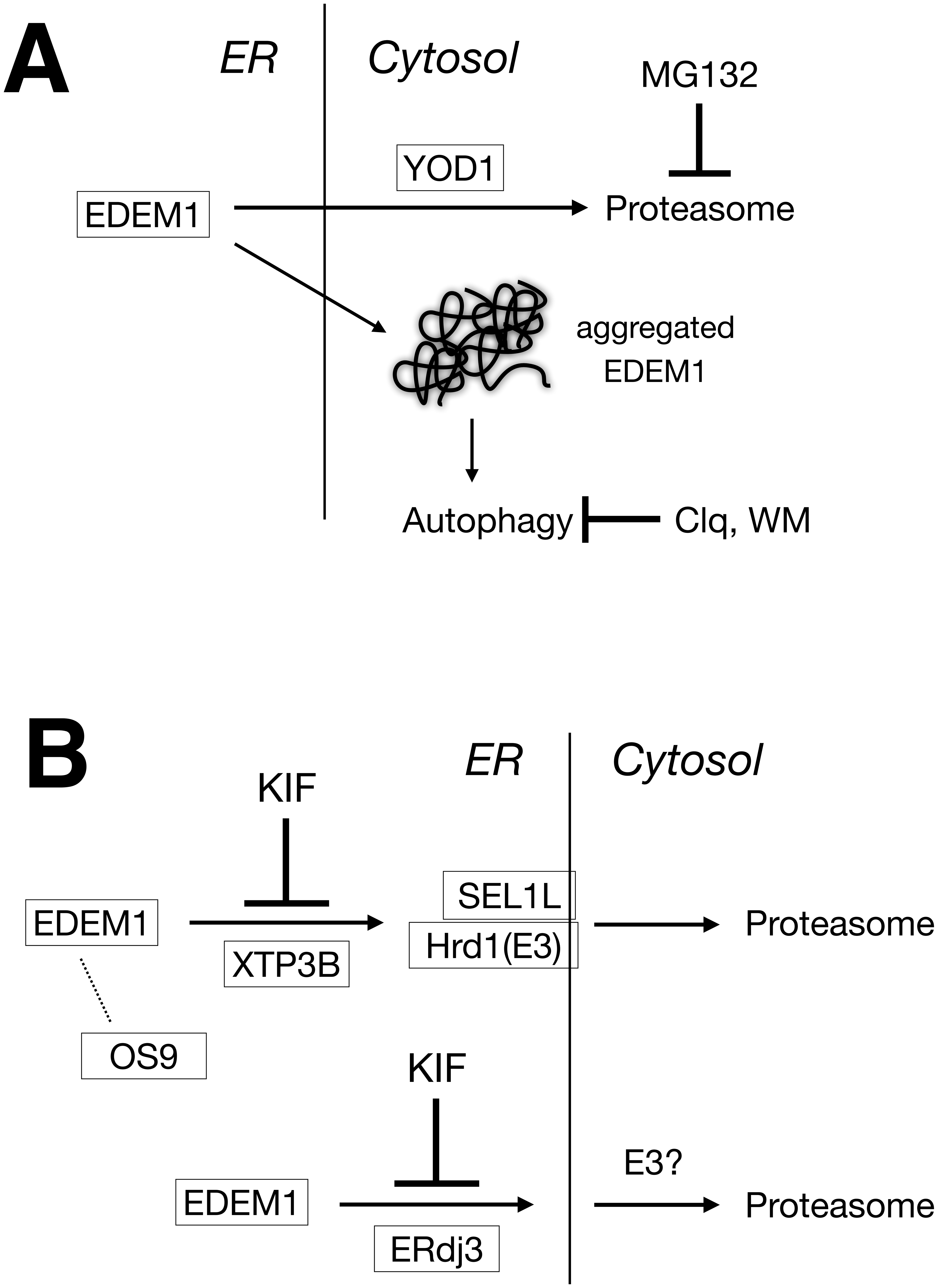
A
ER
Cytosol
MG132
YOD1
EDEM1
Proteasome
aggregated
EDEM1
Autophagy
Clq, WM
B
KIF
ER
Cytosol
SEL1L
EDEM1
Proteasome
Hrd1(E3)
XTP3B
OS9
KIF
E3?
EDEM1
Proteasome
ERdj3

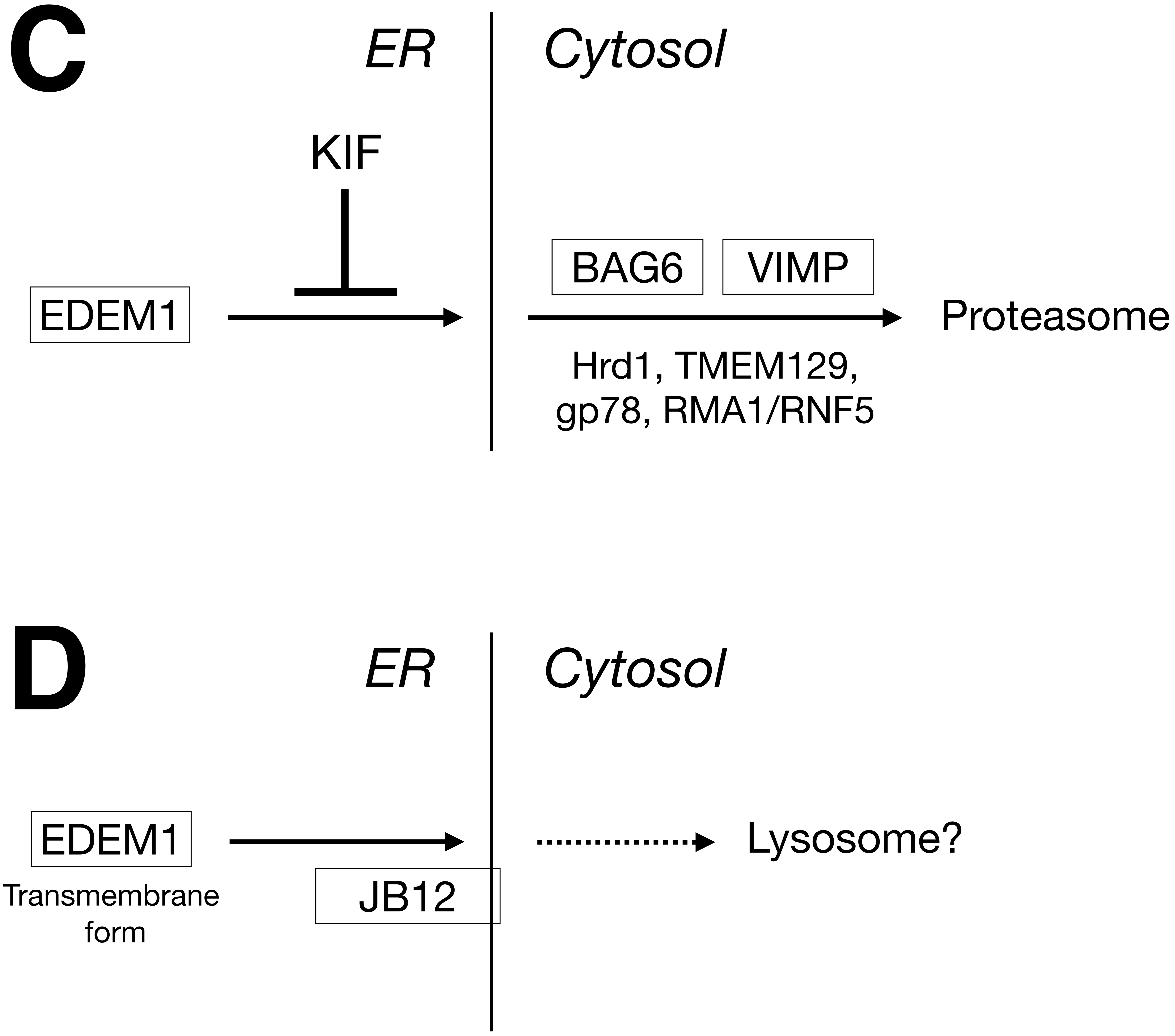

Suuplementary Fig. 12.
The working hypothesis of EDEM1 degradation flows

(A) EDEM1 is degraded by ERAD mediated after polyubiquitination and following deubiquitination by YOD1. Some EDEM1 is aggregated in both the proteasome and lysosomes are down-regulated.

(B) ERAD factors related to mannose trimming are involved in EDEM1 degradation. OS9 associates with EDEM1 but is not involved. ERdj3 also enhances EDEM1 degradation in a KIF-dependent manner via unknown E3 or another pathway.

(C) Cytosolic quality control factors, BAG6 and VIMP, take part in EDEM1 degradation. These are inhibited by KIF in different effectiveness probably due to different E3s and supporting accessory proteins.

(D) JB12 prefers the transmembrane form of EDEM1 for the degradation. Various cytosolic factors, bind to JB12, are involved in EDEM1 clearance.

Supplemental Table 1 Plasmids and primers used in this study.

Plasmids	Source	Primer	Primer sequence (5′-3′)
pCX4-bsr-EDEM1-Flag	Constructed by our laboratory. Ref.		
pXTP3B-mRFP	Donated by Dr. Ikuo Wada (Fukushima Medical University, Japan)		
pVIMP-cfSGFP2	Donated by Dr. Ikuo Wada (Fukushima Medical University, Japan)		
pCI-neo-S-BAG6	Donated by Dr. Hiroyuki Kawahara of (Tokyo Metropolitan University, Japan). Ref.		
pcDNA-SEL1L-HA	Donated by Dr. Nobuko Hosokawa (Kyoto University, Japan). Ref.		
pcDNA-OS9v2-HA	Donated by Dr. Nobuko Hosokawa (Kyoto University, Japan). Ref.		
pcDNA-Hrd1-myc wt	Donated by Dr. Nobuko Hosokawa (Kyoto University, Japan). Ref.		
pcDNA-Hrd1-myc c/s	Donated by Dr. Nobuko Hosokawa (Kyoto University, Japan). Ref.		
pcDNA-mCherry-Ub	Donated by Dr. Akira Kitamura (Hokkaido University, Japan)		
pcDNA-ERdj3 wt-myc	This study (constructed by our laboratory).	ERdj3 RT	GCTAACAACTCCAACCCTCTATATTG
		ERdj3f	GTGAGGAGTGTGTGGAACAGGACCC
		ERdj3r	GTCTGGTGCTGCCGCCTGAGTTTCAAG
pcDNA-ERdj3 H53Q-myc	This study (constructed by our laboratory).	ERdj3 Eco47IIIf	GAACAGGAAGCGCTACAGAGGAACCAT
		ERdj3 EcoRIr	GGACTGCAAGGAGAATTCGAGTGAATAA
pCMV-JB12 wt-HA	This study (constructed by our laboratory).	JB12-HA RT	CAGCCCCATGGCAGGTTTTCTGTCTG
		JB12-HA EcoRIf	CCGCGTTCGAATTCCGCCATGGAATCCAAC
		JB12-HA XhoIr	GGCCCAGGACTCTCGAGGCAGGGAGG
pCMV-JB12 H138Q-HA	This study (constructed by our laboratory).	JB12H138Qf	CCTCAAATTCCAGCCAGACAAGAACCAC
		JB12H138Qr	GCCAGTCTGCGGTAGGCCTTCTTCAG
pCMV-HA-YOD1 wt	This study (constructed by our laboratory).	HA-YOD1 RTr	GGTCAGTGTCAGTAGGTGGCAAGGATC
		HA-YOD1 SalIf	CAACTTCTGTCGACGTTTGGCCCCGC
		HA-YOD1 KpnIr	GGATGTGTGAGGTACCAGGCTTCAACCC
pCMV-HA-YOD1 C160S	This study (constructed by our laboratory).	HA-YOD1 C160Sf	CCCAGCAGACAACTCTAGCCTCTTTACTAGTG
		HA-YOD1 C160Sr	ACCACGGTTCTGGTAAGCACAGGCAAAG

Supplemental Table 2 Antibodies used in this study.

Antibodies	Raised species	Identifier	dilution (WB)	dilution (IS)
BAG6	Mouse	Santa Cruz (sc-365928)	1:2000	
BiP	Rabbit	Donated from Dr. Tetsuro Yamashita, Iwate University, Japan	1:1000	1:100
Calnexin	Rabbit	Donated from Dr. Tetsuro Yamashita, Iwate University, Japan	1:1000	
Calreticulin	Rabbit	Donated from Dr. Tetsuro Yamashita, Iwate University, Japan	1:4000	1:1000
ERdj3	Mouse	Santa Cruz (sc-271240)	1:2000	
ERp57	Rabbit	Donated from Dr. Tetsuro Yamashita, Iwate University, Japan	1:4000	1:1000
Flag	Mouse	Sigma (F1804)	1:4000	1:1000
HA	Rat (monoclonal)	Proteintech (7C9)		1:100
HA	Mouse	MBL (M180-3)	1:4000	
Hsc70	Rat (monoclonal)	Enzo (ADI-SPA-815-D)	1:4000	
HSP70	Mouse	Stressgen (SPA810)	1:4000	
JB12 (DNAJB12)	Rabbit	Sigma (HPA010642)	1:2000	
mRFP	Rabbit	Donated from Dr. Ikuo Wada, Fukushima Medical University, Japan	1:2000	
Myc	Mouse	CST Japan (#2276, 9B11)	1:4000	
S tag	Rabbit	MBL (PM021)	1:2000	
SEL1L	Rabbit	Sigma (S3699)	1:1000	
Ubiquitin (pan)	Rabbit	DAKO (Z0458)	1:1000	
Ubiquitin (K48)	Rabbit (monoclonal)	Millipore (51307)	1:1000	
VIMP (Selenoprotein S)	Mouse	Santa Cruz (sc-365498)	1:1000	
YFP-GST	Rabbit	Donated from Dr. Ikuo Wada, Fukushima Medical University, Japan	1:4000	
YOD1	Rabbit	Proteintech (25370-1-AP)	1:1000	
αTubulin	Mouse	Cedarlane (CLT9002)	1:10000	
anti-Mouse IgG 488	Goat	Molecular Probe (A11029)		1:400
anti-Mouse IgG 555	Goat	Molecular Probe (A21424)		1:400
anti-mouse IgG 647	Goat	Molecular Probe (A21236)		1:400
anti-Rabbit IgG 594	Goat	Molecular Probe (A11037)		1:400
anti-Rat IgG 488	Goat	Molecular Probe (A11006)		1:400
anti-Mouse IgG HRP	Rabbit	Molecular Probe (A9044)	1:5000	
anti-Rabbit IgG HRP	Goat	Molecular Probe (A0545)	1:5000	
